# The Dutch LATER physical outcomes set for self-reported data in survivors of childhood cancer

**DOI:** 10.1007/s11764-020-00880-0

**Published:** 2020-05-03

**Authors:** Nina Streefkerk, Wim J. E. Tissing, Margriet van der Heiden-van der Loo, Elizabeth A. M. (Lieke) Feijen, Eline van Dulmen-den Broeder, Jacqueline J. Loonen, Helena J. H. van der Pal, Cécile M. Ronckers, Hanneke M. van Santen, Marleen H. van den Berg, Renée L. Mulder, Joke C. Korevaar, Leontine C. M. Kremer

**Affiliations:** 1 Department Pediatric Oncology, Amsterdam UMC, Emma Children’s Hospital, University of Amsterdam, Amsterdam, The Netherlands; 2 Princess Máxima Center for Pediatric Oncology, Heidelberglaan 25, 3584 CS Utrecht, The Netherlands; 3 Department of Pediatric Oncology/Hematology, Beatrix Children’s Hospital/University of Groningen/University Medical Center Groningen, Groningen, The Netherlands; 4 Dutch Childhood Oncology Group, Utrecht, The Netherlands; 5 Department of Pediatric Oncology/Hematology, Amsterdam UMC, Vrije Universiteit Amsterdam, Amsterdam, The Netherlands; 6 Department of Hematology, Radboud University Medical Center, Nijmegen, The Netherlands; 7 Department of Pediatric Endocrinology, Wilhelmina Children’s Hospital, University Medical Center, Utrecht, The Netherlands; 8 Netherlands Institute for Health Services Research, Utrecht, The Netherlands

**Keywords:** Childhood cancer survivors, Long-term morbidity, Outcome assessment, Outcome definition

## Abstract

**Purposes:**

Studies investigating self-reported long-term morbidity in childhood cancer survivors (CCS) are using heterogeneous outcome definitions, which compromises comparability and include (un)treated asymptomatic and symptomatic outcomes. We generated a Dutch LATER core set of clinically relevant physical outcomes, based on self-reported data. Clinically relevant outcomes were defined as outcomes associated with clinical symptoms or requiring medical treatment.

**Methods:**

First, we generated a draft outcome set based on existing questionnaires embedded in the Childhood Cancer Survivor Study, British Childhood Cancer Survivor Study, and Dutch LATER study. We added specific outcomes reported by survivors in the Dutch LATER questionnaire. Second, we selected a list of clinical relevant outcomes by agreement among a Dutch LATER experts team. Third, we compared the proposed clinically relevant outcomes to the severity grading of the Common Terminology Criteria for Adverse Events (CTCAE).

**Results:**

A core set of 74 self-reported long-term clinically relevant physical morbidity outcomes was established. Comparison to the CTCAE showed that 36% of these clinically relevant outcomes were missing in the CTCAE.

**Implications for Cancer Survivors:**

This proposed core outcome set of clinical relevant outcomes for self-reported data will be used to investigate the self-reported morbidity in the Dutch LATER study. Furthermore, this Dutch LATER outcome set can be used as a starting point for international harmonization for long-term outcomes in survivors of childhood cancer.

**Electronic supplementary material:**

The online version of this article (10.1007/s11764-020-00880-0) contains supplementary material, which is available to authorized users.

## Introduction

The vast majority of children diagnosed with cancer nowadays will achieve long-term survival [1, 2]. Those childhood cancer survivors (CCS) are a growing, vulnerable group of individuals who are at risk of developing long-term morbidity due to previous treatment for cancer in early stages of life. Knowledge on the burden of long-term morbidity in CCS, its underlying types of health conditions and its risk factors, has been presented in various studies during the past decades [3–5].

In long-term morbidity research in CCS, a broad variety of outcome assessment methods is used. Long-term morbidity outcomes can be assessed by self-reporting via questionnaires [6–24], by medical evaluation during outpatient clinic visits [25–34] or by linkage with existing registries such as national hospital discharge registries [35–39]. Authors often include different types and different numbers of organ systems in their calculations of physical long-term morbidity [6–39]. Also, incidence or prevalence estimates are often reported without describing which health conditions or organ systems were included in these calculations. Definitions of long-term morbidity outcomes also vary, for example, authors reporting on cardiovascular conditions generally report on heart failure, myocardial infarction, and hypertension, but some also include stroke as a cardiovascular condition [10, 14, 17, 18, 36]. While many authors do not grade the severity of the reported long-term morbidity in CCS, others use the Common Terminology Criteria for Adverse Events (CTCAE) [40], either in its original form or an adapted version incorporating specific additional outcomes that authors considered missing [41–43]. This lack of uniformity in types of outcomes, outcome definitions, and outcome grading—even among studies that use similar data ascertainment methods—limits interpretation, comparability, and generalizability of studies investigating the burden of long-term morbidity in CCS. Furthermore, the described outcomes in current studies include asymptomatic and symptomatic outcomes with or without treatment. To get a better insight in the overall burden for survivors, the Dutch LATER questionnaire study would like to evaluate only outcomes that are symptomatic and/or requiring medical treatment.

The aim of this study is to develop a set of self-reported long-term physical outcomes that are clinically relevant for CCS, defined as morbidities with clinical symptoms and/or requiring medical treatment, to investigate the burden of morbidity in the Dutch LATER questionnaire study.

## Methods

### Development of draft outcomes set based on existing questionnaires and input from survivors

Three commonly used questionnaires addressing long-term morbidity in childhood cancer survivors were used for this article: the Dutch Childhood Oncology Group—Long-Term Effects After Childhood Cancer (Dutch LATER) study questionnaire which was used in the Dutch LATER research program [44], the Northern American Childhood Cancer Survivor Study questionnaire [45], and the British Childhood Cancer Survivor Study questionnaire [46]. See Supplementary Tables S1–S3 for the respective items. In long-term morbidity research, the Childhood Cancer Survivor Study questionnaire was used either in its original form [6–8, 10, 12–15, 18, 20, 22, 24, 47–52] or adapted by authors for their own specific study [9, 21, 53]. The questionnaires covered multiple dimensions of late side effects. For this article, we focused on self-reported physical outcomes, covered by the questionnaire sections on medical history and health conditions.

**Fig. 1:**
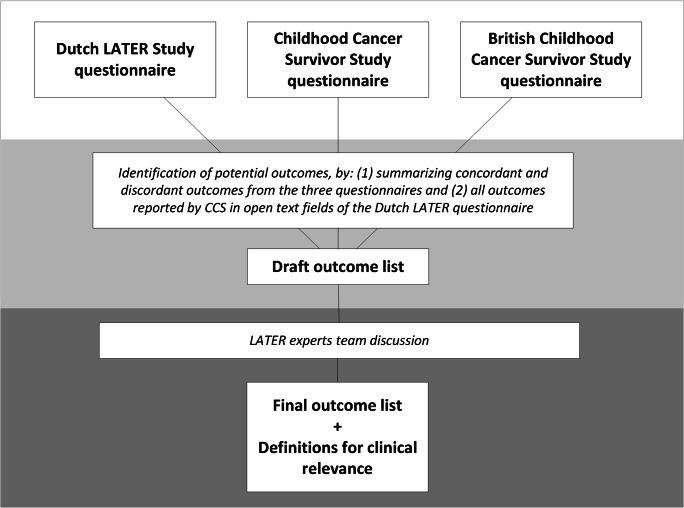
Overview steps followed in the process of development of patient reported outcome list for research for physical long-term morbidity in childhood cancer survivors

The methods of comparing the three long-term morbidity questionnaires and selection of self-reported long-term physical outcomes for CCS are summarized in Fig. 1. We condensed all outcomes from the three questionnaires into 15 categories. All but two were defined per organ system, i.e., conditions of the eye, ear, speech, cardiac, vascular, pulmonary, gastro-intestinal, hepatic, renal and urinary tract, endocrine, musculoskeletal, neurologic conditions, and other conditions. In addition, surgical procedures and malignancies were considered (Supplementary Table S4). We listed the concordances and discordances in outcomes embedded in the three aforementioned questionnaires.

The draft outcome set consisted of a selection of (concordant and discordant) outcomes. Next, we reviewed all health conditions that were reported in the open text fields by CCS participating in the Dutch LATER questionnaire study and added these outcomes to the draft outcome set by outcome category. Temporary or self-limiting morbidities, for example, urinary tract infections, pneumonia, and runner’s knee, were not considered as potential outcomes due to their transient nature and were, therefore, removed from the draft outcome set. Childhood cancer-directed surgeries impacting CCS in later life, for example, limb amputation which results in a lifelong disability or removal of an eye which results in lifelong complications, were added to the draft outcome list. Also, obesity and underweight were added because they were no self-reported outcome in the aforementioned questionnaires.

### Selection of self-reported long-term physical outcomes for CCS

The draft outcome set was reviewed in detail by the Dutch LATER experts team, which comprised a multidisciplinary team of late effects clinicians (pediatric oncology and medical oncology), late effects researchers, a pediatric endocrinologist, and a survivor representative, all of whom are involved in the late effects research. The experts team focused on health conditions that were relevant for childhood cancer survivors, i.e., health conditions that influence their daily life, either by resulting in symptoms or by requiring medical treatment. A proposal for a core outcome set was established by agreement by two authors (N.S. and L.F.), which was discussed by the experts team in a phone meeting. During this meeting, agreement was established regarding a final core set, containing all outcomes deemed relevant for survivors.

Subsequently, for each outcome in the core set, definitions for clinical relevance were established by three authors (N.S., L.F., and L.K.), based on outcome-specific (potential) clinical symptoms and/or (potential) medical treatment. For obesity and underweight in adults, clinical relevance was defined according to the definitions used by the World Health Organization. These definitions were discussed by the experts team by e-mail, until agreement was reached for all clinical relevance criteria.

### Comparison between CTCAE and the new Dutch LATER core outcome set

The CTCAE, originally developed to score acute treatment toxicities [40, 54], is commonly used to grade the severity of outcomes in survivorship studies. This terminology comprises a 5-point grading scale for many adverse events, which are defined as unfavorable and unintended signs, symptoms, or disease, associated with the use of medical treatment. Severity grades rank from 1 (mild, asymptomatic or mild symptoms; clinical or diagnostic observations only; intervention not indicated) to 5 (death related to adverse event) [40]. To gain insight in the agreement between our newly defined outcome set and CTCAE grading, we added the CTCAE grade based on version 4.03 corresponding to our outcome definition for every proposed physical long-term morbidity outcome. Recently, researchers from the St. Jude Lifetime Cohort Study (SJLIFE) adjusted the CTCAE criteria to grade long-term morbidity in their cohort for which data was obtained during clinical assessment using multiple diagnostic modalities. To get insight in concordance between the CTCAE outcomes and the Dutch LATER core outcome set, we compared the different lists of outcomes.

## Results

### Selection of self-reported long-term physical outcomes of clinical relevance

The process of selection of self-reported clinically relevant physical long-term physical outcomes, as displayed in Fig. 1, resulted in a core outcome set consisting of 74 proposed outcomes. The experts team decided on re-categorizing surgical procedures within their respective organ system and did not consider conditions of speech as clinically relevant. Therefore, the 15 initial outcome categories were re-categorized into 13 proposed main organ system categories: conditions of the eye, ear, cardiac, vascular, respiratory, gastro-intestinal, hepatobiliary tract, renal and urinary tract, endocrine, musculoskeletal, nervous system conditions, other conditions, and neoplasms (see Table 1).

**Table 1:** Core set of self-reported long-term physical outcomes of clinical relevance for childhood cancer survivors

	Self-reported long-term physical outcome	Definition of clinical relevance
Eye disorders	Cataract	Cataract of at least one eye treated with surgery
	Blindness	Blindness of at least one eye
	Eye removal	Status after removal of at least one eye
Ear	Hearing loss	Hearing loss of at least one ear, requiring a hearing aid
conditions	Deafness	Deafness of at least one ear
Cardiac conditions	Heart failure	Heart failure with clinical symptoms, with at least one of the following criteria: 1. Requiring medication (ACE inhibitors, beta-blockers, mineralocorticoid receptor antagonists, aldosterone receptor antagonists, diuretics, angiotensin II blockers, digoxin) 2. Requiring devices (CRT-P or CRT-D, pacemaker, ICD, LVAP, cardiac reduction surgery)
	Ischemia	Cardiac ischemia with clinical symptoms requiring intervention (angioplasty, stent, coronary bypass graft)
	Pericarditis	Pericarditis with clinical symptoms, with at least one of the following criteria: 1. Life-threatening consequences (hemodynamic comprise, tamponade)2. Requiring surgical intervention (pericardiectomy)
	Valvular disease	Valvular disease with clinical symptoms, with at least one of the following criteria: 1. Requiring medication (ACE inhibitors, calcium channel blockers, beta-blockers, enalapril, diuretics, digoxin) 2. Requiring valve replacement or valvuloplasty
	Arrhythmia	Arrhythmia with clinical symptoms, with at least one of the following criteria: 1. Requiring medication (beta-blockers, digoxin, calcium channel blockers, amiodarone, sotalol, flecainide, propafenone, electrolytes, anti-thrombines, anti-platelets, N-3 fatty acid and lipids) 2. Requiring device or surgical intervention (ICD, pacemaker, CRT-P, CRT-D, ablation, antiarrhythmic surgery, cardioversion)
	Heart transplantation	Status after heart transplantation
Vascular conditions	Hypertension	Hypertension, requiring antihypertensive medication (ACE inhibitors, beta blockers, diuretics, calcium antagonists, angiotensin II antagonists, alfa blockers)
	Thrombosis	Thrombosis or a thromboembolic event, with at least one of the following criteria: 1. Requiring chronic treatment with antithrombotic agents 2. Requiring surgical intervention
	Aneurysm	The presence of an aneurysm (confirmed by medical imaging), requiring surgical intervention
Respiratory conditions	Obstructive pulmonary disease	Pulmonary obstructive disease (i.e., asthma, COPD, chronic bronchitis), with clinical symptoms, with at least one of the following criteria: 1. Requiring chronic medication (beclometason, fluticasone proprionate, ciclesonide, salmeterol, beclomethasone/formoterol, budesonide/formoterol, salmeterol/estril, montelukast) 2. Requiring chronic oxygen treatment *Only intermittent therapy with acute bronchodilators is not defined as clinically relevant
	Decreased pulmonary function	Decreased pulmonary function confirmed by spirometry function, which results in limitations in daily life on participation level (i.e., due to the pulmonary condition unable to function in work, hobbies, household, or social circumstances) *Asymptomatic decreased lung function without symptoms detected during routine screening is not defined as clinically relevant
	Pulmonary resection	Status after surgery to remove (part of a) lung after which symptoms of decreased pulmonary function are present
	Pulmonary transplantation	Transplantation of one or more lungs after which symptoms of decreased pulmonary function are present
	Other pulmonary conditions	Other pulmonary conditions (including bullae, pulmonary edema, pleuritis) with clinical symptoms, confirmed by clinical evaluation, with at least one of the following criteria: 1. Requiring medical treatment with medication or surgery 2. Resulting in limitations in daily life on participation level (due to the pulmonary condition unable to function in work, hobbies, household or social circumstances)
Gastro-intestinal	Gastroesophageal reflux disease	Gastroesophageal reflux disease, with clinical symptoms, requiring anti acid medication
	Inflammatory bowel disease	Inflammatory bowel disease (i.e., Crohn and Colitis ulcerosa) with clinical symptoms, with at least one of the following criteria: 1. Requiring treatment with immunosuppressive medication 2. Requiring surgical intervention
	Other gastrointestinal conditions	Gastro-intestinal health conditions, not otherwise specified, with clinical symptoms, causing mechanical problems (i.e., adhesions, ileus, stenosis, stoma), with at least one of the following criteria: 1. Requiring chronic tube feeding 2. Requiring chronic total parenteral feeding 3. Requiring surgical intervention 4. The presence of a stoma 5. The removal of (part of the) jaw
Hepatobiliary conditions	Hepatitis	Chronic infection with hepatitis B or C, with at least one of the following criteria: 1. Requiring at least one of the listed medication (interferon or antiviral medication) 2. Resulting in liver cirrhosis
	Hemochromatosis	Hemochromatosis (iron overload), with clinical symptoms, with at least one of the following criteria: 1. Requiring treatment with phlebotomy or erythrocytopheresis 2. Requiring iron lowering medication
	Liver cirrhosis	Cirrhosis of the liver with clinical symptoms
	Liver transplantation	Status after liver transplantation
	Cholecystectomy	Status after cholecystectomy
Renal and urinary tract	Tubular dysfunction	The presence of renal tubular dysfunction with clinical symptoms, resulting in electrolyte imbalance requiring medication
conditions	Proteinuria	Proteinuria confirmed by urine analysis, requiring treatment with medication (ACE inhibitors, thiazide diuretics)
	Chronic kidney disease	Renal insufficiency with clinical symptoms, requiring medical treatment with at least one of the following: 1. Antihypertensive drugs (ACE inhibitors, angiotensin II antagonists, diuretics) 2. Medication for proteinuria (ACE inhibitors or thiazide diuretics) 3. Medication for the prevention of cardiovascular complications (statins) 4. Medication for anemia (EPO) 5. Medication for osteodystrophia (phosphate binding medicine, active vitamin D) 6. Medication for electrolyte deficiencies/tubular dysfunction 7. Dialysis 8. Renal transplantation
	Urinary tract obstruction	Urinary tract obstruction with clinical symptoms, requiring surgical intervention
	Nephrectomy	Status after the removal of at least one kidney
	Renal transplantation	Status after transplantation of one (or more) kidney(s)
	Other conditions of kidney and urinary tract	Other conditions of kidney and urinary tract with clinical symptoms, including: 1. The presence of an urine stoma 2. Incontinence, requiring surgical intervention 3. The need for structural catheterization 4. Dialysis 5. Removal of bladder 6. Elevated uric acid treated with chronic medication
Endocrine conditions	Adrenal insufficiency^B^	Adrenal insufficiency with clinical symptoms and confirmed by laboratory testing, requiring hormonal treatment (glucocorticoids, mineralocorticoids)
	Hypercortisolism	Hypercortisolism (Cushing’s disease) with clinical symptoms and confirmed by laboratory testing, with at least one of the following criteria: 1. Requiring surgical intervention 2. Requiring radiation therapy 3. Requiring post-treatment substitution therapy (hydrocortisone)
	Hypothyroidism^B^	Hypothyroidism with clinical symptoms and confirmed by laboratory testing requiring treatment with chronic medication (levothyroxine)
	Hyperthyroidism	Hyperthyroidism with clinical symptoms and confirmed by laboratory testing, with at least one of the following criteria: 1. Requiring iodine treatment (radioactive) 2. Requiring surgical intervention (i.e., (hemi)thyroidectomy) 3. Requiring medication (i.e., thyreostatics or thyroid suppletion therapy for iatrogenic hypothyroidism)
	Estrogen deficiency^B^	Estrogen deficiency with clinical symptoms and confirmed by laboratory testing, with at least one of the following criteria: 1. Requiring treatment with transdermal estrogen 2. Requiring chronic medication (oral estrogen)
	Testosterone deficiency^B^	Testosterone deficiency with clinical symptoms and confirmed by laboratory testing, requiring treatment with: Testosterone
	Growth hormone deficiency^B^	Growth hormone deficiency with clinical symptoms and confirmed by laboratory testing, with at least one of the following criteria: 1. Requiring medical treatment with growth hormone 2. For which growth hormone treatment was indicated, but the treating physician and/or parents decided not to start this treatment because of medical contra-indications
	Hypoparathyroidism	Hypoparathyroidism with clinical symptoms and confirmed by laboratory testing, with at least one of the following criteria: 1. Requiring calcium suppletion 2. Requiring active vitamin D3 (calcitriol or etalpha)
	Hyperparathyroidism	Hyperparathyroidism with clinical symptoms and confirmed by laboratory testing, requiring surgical intervention
	Prolactinoma	Prolactinoma with clinical symptoms and confirmed by laboratory testing, with at least one of the following criteria: 1. Requiring treatment with dopamine agonists 2. Requiring surgical treatment
	Polycystic ovarian syndrome	The presence of polycystic ovarian syndrome with clinical symptoms, confirmed by imaging
	Precocious puberty	Early puberty, that has been, or is currently treated with medication (puberty inhibiting medicine, i.e., GnRH analogues)
	Pubertas tarda	Late puberty, that has been or is currently treated with medication (sex steroids)
	Pituitary deficiency	Pituitary deficiency, with clinical symptoms and confirmed by laboratory testing, with at least one of the following criteria: 1. Requiring growth hormone treatment 2. Requiring thyroid hormone treatment 3. Requiring hydrocortisone treatment 4. Requiring sex hormone treatment 5. Requiring desmopressin treatment
	Pituitary surgery	Status after surgery to the pituitary gland
	Obesity	The presence of obesity according to the World Health Organization’s standardized definition of obesity for adults: BMI > 30, or for children > + 2 SDS in corrected for age and sex according to Dutch normative data^A^
	Underweight	The presence of underweight according to the World Health Organization’s standardized definition of underweight for adults: BMI < 18.5, or for children < − 2 SDS corrected for age and sex according to Dutch normative data^A^
	Diabetes mellitus	Diabetes mellitus with confirmed by laboratory testing with at least one of the following criteria: 1. Requiring treatment with oral antidiabetic agents 2. Requiring treatment with intramuscular or intravenous insulin
	Diabetes insipidus^B^	Diabetes insipidus with clinical symptoms and confirmed by laboratory testing, requiring treatment with medication (desmopressin)
	Thyroidectomy	Status after (partial) thyroidectomy, after which medication use (levothyroxine) is required
	Adrenal gland removal	Status after the removal of one or two adrenal gland(s)
	Ovariectomy	Status after the removal of one or more ovaria
	Orchidectomy	Status after the removal of one or more testes
Nervous system conditions	Cerebrovascular accident—hemorrhagic	Intracranial hemorrhage with clinical symptoms and confirmed by imaging, with at least one of the following criteria: 1. Requiring surgical intervention 2. Requiring medication (antihypertensive drugs)
	Cerebrovascular accident—ischemic	Intracerebral infarction with clinical symptoms and confirmed by imaging, requiring treatment with medication (acetylsalicylic acid, dipyridamole, statins, or antihypertensive agents)
	Transient ischemic attack	The presence of a transient ischemic attack (duration < 24 h) with clinical symptoms, requiring treatment with medication (acetylsalicylic acid, dipyridamole, statins, or antihypertensive agents)
	Epilepsy	Epilepsy with clinical symptoms and confirmed by electro-encephalography, requiring treatment with medication (carbamazepine, lamotrigine, levetiracetam, oxcarbezepine, valproate, clonazepam, phenytoin, gabapentin, lacosamide, perampanel, pregabalin, topiramate, zonisamide, clonazepam)
	Headache	Headache (migraine, cluster headache) resulting in clinical symptoms treated with at least one of the following criteria: 1. Requiring treatment with beta blockers, anti-epileptic medication, flinarizine, pizotifen, methysergide, or candesartan (migraine) 2. Requiring treatment with verapamil, lithium carbonate, methysergide, pizotifen, ergotamine, or prednisone (cluster headache)
	Hydrocephalus	The presence of hydrocephalus, requiring surgical intervention
	Other neurological conditions	The presence of other neurologic conditions, with clinical symptoms, including facialis paresis, spinal cord injury, (spastic) paresis, loss of strength, disturbance of equilibrium, coordination problems, vertigo, acquired brain injury, tremor, parkinsonism, ataxia)
Musculoskeletal conditions	Amputation	Status after the amputation of a (part of a) limb, excluding fingers and toes
	Deformities	The presence of at least one of the following major deformities (scoliosis, kyphosis, lordosis, or spondylolisthesis) with clinical symptoms
	Osteoporosis	Osteoporosis confirmed with a DEXA scan, requiring treatment with chronic medication (bisphosphonates, estrogen receptor modulators, or parathyroid hormone)
	Other musculoskeletal conditions	At least one of the following conditions with clinical symptoms: arthritis (bacterial, gout, reactive, rheumatoid arthritis), arthrosis, osteonecrosis, epiphysiolysis, with at least one of the following criteria: 1. Requiring medication (allopurinol, benzbromaron, leflunomide, methotrexate, sulfasalazine, infliximab, adalimumab, etanercept, certolizumab, anti-IL1, anti-CD80, anti-CD86, aurothiomalaat, ciclosporin, hydroxychloroquinine, cyclophosphamide) 2. Requiring therapy using intra-articular injection(s) 3. Requiring joint replacement surgery 4. Requiring arthrodesis surgery
Neoplasms	Malignant neoplasms	Malignant neoplasms of any kind
Other conditions	Dermatological conditions	Dermatological conditions with clinical symptoms and that require systematic treatment
	Hysterectomy	Status after the removal of the uterus
	Prostatectomy	Status after the removal of the prostate
	Mastectomy	Status after the removal of one or more breast(s)
	Splenectomy	Status after splenectomy

^A^In this study we used Dutch population-based normative data for children below 18 years. For international harmonization, we recommend using Child Growth Standards from the World Health Organization

^B^When this hormonal deficiency is the result of pituitary dysfunction, it is categorized separately as “pituitary deficiency”

### Agreement between the newly defined core outcome set and the CTCAE grading

For each outcome, the minimum CTCAE grades that correspond with our criteria for clinical relevance are shown in Supplementary Table S5. In all, 27 out of 74 (36%) outcomes cannot be graded according to CTCAE because they are not present in the CTCAE as a separate entity. This group of outcomes can be categorized into three subgroups. First, it comprised certain surgeries of which the LATER experts team agreed upon clinical relevance (*n* = 18), because they influence CCS’s daily life either by having medical consequences (e.g., splenectomy or organ transplantations) or by having cosmetic consequences (e.g., eye enucleation or limb amputation). Second, it comprised blindness and deafness, which are included in the CTCAE not as a specific outcome but as grading scale for several specific other eye and ear/nose/throat outcomes. The LATER experts team agreed that regardless of the underlying pathophysiological mechanism, blindness and deafness were both clinical relevant outcomes that should be included in the core outcome set. Third, specific outcomes that were not present as separate entities in the CTCAE were reported by CCS in the Dutch LATER questionnaire and were perceived as clinically relevant by the experts team (*n* = 7): aortic aneurysm, liver cirrhosis, tubular dysfunction of the kidneys, prolactinoma, polycystic ovarian syndrome, underweight, and pituitary dysfunction.

Of the remaining 48 conditions, 11 (15%) fulfilled the definition for conditions with a CTCAE grade 3, that is, severe or medically significant but not immediately life-threatening. For 27 (36%) conditions, our criteria for clinical relevance corresponded with a CTCAE grade 2, moderate severity. For nine (12%) conditions (decreased pulmonary function, proteinuria, chronic kidney disease, precocious puberty, diabetes mellitus, ischemic cerebrovascular accident, transient ischemic attack, epilepsy, and headache), it was not possible to define the corresponding CTCAE grade for our established clinical relevance criteria, because additional clinical information was needed for CTCAE-based grading. Comparison to the SJLIFE-based grading showed that 34 conditions from our core set were not present in SJLIFE (46%) and additional information was needed for grading of 5 conditions (7%). A total of 23 clinically relevant conditions corresponded with SJLIFE grade 2 (31%) and two clinically relevant conditions (adrenal insufficiency and growth hormone deficiency) corresponded with SJLIFE grade 1 (3%).

## Discussion

We present a proposal for a core set of 74 self-reported long-term physical outcomes of clinical relevance in survivors of childhood cancer. By comparison of existing survivorship questionnaires and by reviewing every specific morbidity reported by CCS in the open text fields in our Dutch nationwide questionnaire study, we followed an innovative method which focuses on outcomes that are clinically relevant for the survivor, due to the fact that its presence influences daily life. Our outcome set will be used for investigating the burden of long-term morbidity in the Dutch LATER questionnaire study. This set can also be used for international harmonization of a uniform core outcome set for long-term morbidity in CCS, to facilitate worldwide collaboration in late effects research.

Compared with other grading scales used for long-term morbidity research in CCS, the newly developed Dutch LATER core outcome set differs on three important key points. First, this core outcome set was designed with the single purpose of investigating self-reported long-term morbidity in childhood cancer survivors, by combining existing questionnaires and outcomes reported by survivors. Second, we selected outcomes describing morbidity with clinical symptoms or requiring medical treatment, the so-called clinically relevant outcomes. Third we included outcomes where the treatment for childhood cancer caused direct damage that had persistent impact for the survivor also in later life, for example, limb amputation which results in a lifelong disability or removal of an eye which results in lifelong complications. Because the CTCAE criteria were originally designed for grading acute adverse events during adult cancer trials [54], the current CTCAE version 4.03 [40] does not cover the complete spectrum of long-term morbidity that CCS might encounter [42]. Several authors have already stated that relevant outcomes were missing for CCS and use adapted versions [41–43]. Comparison of our core set of long-term self-reported physical outcomes to the commonly used CTCAE showed that 36% of the outcomes were not present in the CTCAE. Moreover, CTCAE does not incorporate self-reported data to assess long-term morbidity [42]. For nine out of the 48 conditions that were present in the CTCAE, we could not perform severity grading because detailed additional clinical information was needed for appropriate grading, which was not available from current questionnaires and is often too complicated to directly ask patients in a questionnaire. Although often only health conditions grade 3 and higher are included when studying severe physical long-term morbidity in CCS, our results show that many grade 2 conditions will have consequences for a survivor because of symptoms or needed treatment. From our core outcome set, up to 27 clinically relevant outcomes corresponded with CTCAE grade 2, for example, several endocrine deficiencies that require chronic medication use, and would have been missed in such studies. Comparison to the SJLIFE adapted CTCAE for grading of clinically ascertained data showed that more of our core outcomes were missing and that 24 clinically relevant conditions corresponded to grade 2 or even grade 1. Hence, our results support previous authors, concluding that the CTCAE in its current form is not optimal to grade severity of (self-reported) long-term physical morbidity outcomes for CCS [41–43]. To our knowledge, this is the first comprehensive proposal to define a core outcome set for self-reported long-term physical outcomes in CCS. A strength of this study is that we focused on clinical relevance for CCSA and a limitation is that we were not yet able to incorporate the prioritization of outcomes by survivors. This can be the focus of future research. Also, because the purpose of this core outcome set was facilitating the investigation of physical long-term morbidity in the Dutch LATER cohort, the proposed outcome definitions reflect the agreement among the Dutch LATER experts team only. To overcome any subjectivity in outcomes used by various childhood cancer survivorship research groups, we advocate international harmonization of a core outcome set for physical long-term morbidity in childhood cancer survivors. A uniform global core outcome set is highly needed to enable comparison of future long-term morbidity studies, to uniformly evaluate survivorship care and to facilitate collaboration within survivorship research. The International Guideline Harmonization Group [55] started an initiative to develop a harmonized outcome set by a Delphi method. This will facilitate international collaboration and data pooling.

In conclusion, we propose a Dutch LATER core set of self-reported long-term physical outcomes of clinical relevance for CCS that will be used to investigate the burden of long-term morbidity in childhood cancer survivors from the Dutch LATER questionnaire study. We advocate to start international discussion and research to harmonize long-term physical morbidity outcomes that are clinically relevant for CCS.

## Electronic supplementary material

ESM 1 (DOC 1.36 mb)
