# Supplementary material for: The Dutch LATER physical outcomes set for self-reported data in survivors of childhood cancer
Source: J Cancer Surviv. 2020 May 3;14(5):666–76. doi: 10.1007/s11764-020-00880-0 (PMC7473963; doi:10.1007/s11764-020-00880-0)
Supplement: Supplementary file 1 — (DOC 1.36 mb) [file 11764_2020_880_MOESM1_ESM.doc]

**Supplementary Table S1.** Translation of the DCOG-LATER questionnaire section on medical information

This table provides an English translation of all the questions in the DCOG-LATER questionnaire, addressing physical long-term morbidity (question B1 – B7).

| **Question** | **Answer options** |
| --- | --- |
| **B1. You have been treated in childhood for childhood cancer or a related condition. Did you afterwards get another form of cancer, leukemia or a tumor, or are you currently diagnosed with it?** | Yes/No |
| - Yes/No | Yes/No |
| **B2. If yes, please report the organ/body part, type of tumor and age at diagnosis or year of diagnosis.** |  |
| - Organ/Body part | Open text field |
| - Type of tumor | Open text field |
| - Age at diagnosis or year of diagnosis | Open text field |
| **B3. Please fill in in the diagram below whether you have had one of the following conditions** |  |
| **If yes, please note age at diagnosis or year of diagnosis and whether you use medication for this condition at the moment.**  ***If you currently use medication for this condition, please note the name of the medication and the age or year in which you started using it in question B4.*** |  |
| a. Myocardial infarction |  |
| - Have you had this condition or do you currently have this condition? | Yes/No |
| - What was the age at diagnosis or year of diagnosis? | Open text field |
| - Are you currently using medication for this condition? | Yes/No |
| b. Angina pectoris (during exercise or in rest) |  |
| - Have you had this condition or do you currently have this condition? | Yes/No |
| - What was the age at diagnosis or year of diagnosis? | Open text field |
| - Are you currently using medication for this condition? | Yes/No |
| c. Valvular condition |  |
| - Have you had this condition or do you currently have this condition? | Yes/No |
| - What was the age at diagnosis or year of diagnosis? | Open text field |
| - Are you currently using medication for this condition? | Yes/No |
| d. Pericarditis |  |
| - Have you had this condition or do you currently have this condition? | Yes/No |
| - What was the age at diagnosis or year of diagnosis? | Open text field |
| - Are you currently using medication for this condition? | Yes/No |
| e. Cardiomyopathy |  |
| - Have you had this condition or do you currently have this condition? | Yes/No |
| - What was the age at diagnosis or year of diagnosis? | Open text field |
| - Are you currently using medication for this condition? | Yes/No |
| f. Heart failure |  |
| - Have you had this condition or do you currently have this condition? | Yes/No |
| - What was the age at diagnosis or year of diagnosis? | Open text field |
| - Are you currently using medication for this condition? | Yes/No |
| g. Arrythmia |  |
| - Have you had this condition or do you currently have this condition? | Yes/No |
| - What was the age at diagnosis or year of diagnosis? | Open text field |
| - Are you currently using medication for this condition? | Yes/No |
| h. Congenital heart disease |  |
| - Have you had this condition or do you currently have this condition? | Yes/No |
| - Open text field for reporting the specific condition | Open text field |
| - What was the age at diagnosis or year of diagnosis? | Open text field |
| - Are you currently using medication for this condition? | Yes/No |
| i. Other heart disease |  |
| - Have you had this condition or do you currently have this condition? | Yes/No |
| - Open text field for reporting the specific condition | Open text field |
| - What was the age at diagnosis or year of diagnosis? | Open text field |
| - Are you currently using medication for this condition? | Yes/No |
| k. Stroke (cerebrovascular accident/cerebral infarction/cerebral hemorrhage) |  |
| - Have you had this condition or do you currently have this condition? | Yes/No |
| - What was the age at diagnosis or year of diagnosis? | Open text field |
| - Are you currently using medication for this condition? | Yes/No |
| l. Transient ischemic attack |  |
| - Have you had this condition or do you currently have this condition? | Yes/No |
| - Open text field for reporting the specific condition | Open text field |
| - What was the age at diagnosis or year of diagnosis? | Open text field |
| - Are you currently using medication for this condition? | Yes/No |
| m. Vascular conditions |  |
| - Have you had this condition or do you currently have this condition? | Yes/No |
| - Open text field for reporting the specific condition | Open text field |
| - What was the age at diagnosis or year of diagnosis? | Open text field |
| - Are you currently using medication for this condition? | Yes/No |
| n. Conditions leading to enhanced coagulation (thrombosis) |  |
| - Have you had this condition or do you currently have this condition? | Yes/No |
| If yes: which condition? |  |
| - - Protein C deficiency | Tick box |
| - - - What was the age at diagnosis or year of diagnosis? | Open text field |
| - - - Are you currently using medication for this condition? | Yes/No |
| - - Protein S deficiency | Tick box |
| - - - What was the age at diagnosis or year of diagnosis? | Open text field |
| - - - Are you currently using medication for this condition? | Yes/No |
| - - Factor V Leiden mutation | Tick box |
| - - - What was the age at diagnosis or year of diagnosis? | Open text field |
| - - - Are you currently using medication for this condition? | Yes/No |
| - - Other | Tick box |
| - - - Open text field for reporting the specific condition | Open text field |
| - - - What was the age at diagnosis or year of diagnosis? | Open text field |
| - - - Are you currently using medication for this condition? | Yes/No |
| n. Hypertension |  |
| - Have you had this condition or do you currently have this condition? | Yes/No |
| - What was the age at diagnosis or year of diagnosis? | Open text field |
| - Are you currently using medication for this condition? | Yes/No |
| o. Hypercholesterolemia |  |
| - Have you had this condition or do you currently have this condition? | Yes/No |
| - What was the age at diagnosis or year of diagnosis? | Open text field |
| - Are you currently using medication for this condition? | Yes/No |
| p. Gastro-intestinal problems |  |
| - Have you had this condition or do you currently have this condition? | Yes/No |
| - What was the age at diagnosis or year of diagnosis? | Open text field |
| - Are you currently using medication for this condition? | Yes/No |
| q. Pulmonary conditions |  |
| - Have you had this condition or do you currently have this condition? | Yes/No |
| - Open text field for reporting the specific condition | Open text field |
| - What was the age at diagnosis or year of diagnosis? | Open text field |
| - Are you currently using medication for this condition? | Yes/No |
| r. Period of cough with a duration of over 6 weeks |  |
| - Have you had this condition or do you currently have this condition? | Yes/No |
| - Are you currently using medication for this condition? | Yes/No |
| s. Infections of the respiratory tract over 3 times a year |  |
| - Have you had this condition or do you currently have this condition? | Yes/No |
| - Are you currently using medication for this condition? | Yes/No |
| t. Urinary tract infections, accompanied by fever (pyelonephritis) |  |
| - Have you had this condition or do you currently have this condition? | Yes/No |
| - What was the age at diagnosis or year of diagnosis? | Open text field |
| - If yes: how often? |  |
| - - - 1 time | Tick box |
| - - - 2-5 times | Tick box |
| - - - Over 5 times | Tick box |
| u. Other kidney problems (for example malfunction of kidneys, kidney stones, proteinuria, cysts) |  |
| - Have you had this condition or do you currently have this condition? | Yes/No |
| - Open text field for reporting the specific condition | Open text field |
| - What was the age at diagnosis or year of diagnosis? | Open text field |
| - Are you currently using medication for this condition? | Yes/No |
| v. Adrenal problems |  |
| - Have you had this condition or do you currently have this condition? | Yes/No |
| - Open text field for reporting the specific condition | Open text field |
| - What was the age at diagnosis or year of diagnosis? | Open text field |
| - Are you currently using medication for this condition? | Yes/No |
| w. Liver problems, specified:…….. |  |
| - Have you had this condition or do you currently have this condition? | Yes/No |
| - Open text field for reporting the specific condition | Open text field |
| - What was the age at diagnosis or year of diagnosis? | Open text field |
| - Are you currently using medication for this condition? | Yes/No |
| x. Musculoskeletal problems (for example arm/leg/elbow/knee) |  |
| - Have you had this condition or do you currently have this condition? | Yes/No |
| - Open text field for reporting the specific condition | Open text field |
| - What was the age at diagnosis or year of diagnosis? | Open text field |
| - Are you currently using medication for this condition? | Yes/No |
| y. Diabetes Mellitus |  |
| - Have you had this condition or do you currently have this condition? | Yes/No |
| - What was the age at diagnosis or year of diagnosis? | Open text field |
| - Are you currently using medication for this condition? | Yes/No |
| z. Seizures |  |
| - Have you had this condition or do you currently have this condition? | Yes/No |
| - What was the age at diagnosis or year of diagnosis? | Open text field |
| - Are you currently using medication for this condition? | Yes/No |
| aa. Diagnosed with cataract |  |
| - Have you had this condition or do you currently have this condition? | Yes/No |
| - What was the age at diagnosis or year of diagnosis? | Open text field |
| - Are you currently using medication for this condition? | Yes/No |
| bb. Using a hearing aid |  |
| - Have you had this condition or do you currently have this condition? | Yes/No |
| - What was the age at diagnosis or year of diagnosis? | Open text field |
| - Are you currently using medication for this condition? | Yes/No |
| cc. Ringing in the ears |  |
| - Have you had this condition or do you currently have this condition? | Yes/No |
| - What was the age at diagnosis or year of diagnosis? | Open text field |
| - Are you currently using medication for this condition? | Yes/No |
| dd. Experiencing (or experienced) reduced growth (short stature) |  |
| - Have you had this condition or do you currently have this condition? | Yes/No |
| - What was the age at diagnosis or year of diagnosis? | Open text field |
| - Are you currently using medication for this condition? | Yes/No |
| ee. Impaired thyroid function (hypothyroidism) |  |
| - Have you had this condition or do you currently have this condition? | Yes/No |
| - What was the age at diagnosis or year of diagnosis? | Open text field |
| - Are you currently using medication for this condition? | Yes/No |
| ff. Increased thyroid function (hyperthyroidism) |  |
| - Have you had this condition or do you currently have this condition? | Yes/No |
| - What was the age at diagnosis or year of diagnosis? | Open text field |
| - Are you currently using medication for this condition? | Yes/No |
| gg. Thyroid nodule | Yes/No |
| - Have you had this condition or do you currently have this condition? | Yes/No |
| - What was the age at diagnosis or year of diagnosis? | Open text field |
| - Are you currently using medication for this condition? | Yes/No |
| hh. Other thyroid condition |  |
| - Have you had this condition or do you currently have this condition? | Yes/No |
| - Open text field for reporting the specific condition | Open text field |
| - What was the age at diagnosis or year of diagnosis? | Open text field |
| - Are you currently using medication for this condition? | Yes/No |
| ii. Other problem with hormonal regulation |  |
| - Have you had this condition or do you currently have this condition? | Yes/No |
| - Open text field for reporting the specific condition | Open text field |
| - What was the age at diagnosis or year of diagnosis? | Open text field |
| - Are you currently using medication for this condition? | Yes/No |
| jj. Other health condition |  |
| - Have you had this condition or do you currently have this condition? | Yes/No |
| - Open text field for reporting the specific condition | Open text field |
| - What was the age at diagnosis or year of diagnosis? | Open text field |
| - Are you currently using medication for this condition? | Yes/No |
| **B4. Please note which medicines you are currently using for the conditions listed in question B3 and at what age or in which year you started taking them?**  ***If you do not know the name of the medicine, please enter the name of the disorder from B3 followed by "don't know".*** |  |
| - Medication name (room for multiple answers) | Open text field |
| - For which health condition | Open text field |
| - Start use: age or year | Open text field |
| **B5. Are you currently using other medicines or injections in addition to the medicines that you may have mentioned above? For example, painkillers that you use more than once a week (eg aspirin, paracetamol, Ibuprofen) or hormones.** |  |
| - Yes/No | Yes/No |
| **B6. If yes, which medication do you use and at what age or in which year did you start taking them?** |  |
| - Medication name | Open text field |
| - Start use: age or year | Open text field |
| **B7. Have you ever undergone one or more of the following treatment(s) or surgical procedure(s)?** |  |
| - Did you ever undergo a replacement of a heart valve? | Yes/No |
| - Did you undergo this surgery? | Yes/No |
| - What was the age at surgery or year of surgery? | Open text field |
| - Did you ever undergo any other heart surgery (including heart catheterization)? |  |
| - Did you undergo this surgery? | Yes/No |
| - Open text field for reporting the specific type of surgery | Open text field |
| - What was the age at surgery or year of surgery? | Open text field |
| - Did you ever have a pacemaker/ICD? |  |
| - Did you undergo this surgery? | Yes/No |
| - What was the age at surgery or year of surgery? | Open text field |
| - Have you ever had an organ transplantation? |  |
| - Did you undergo this surgery? | Yes/No |
| - Open text field for reporting which organ | Open text field |
| - What was the age at surgery or year of surgery? | Open text field |
| - Have you ever had an organ/arm/leg removed? |  |
| - Did you undergo this surgery? | Yes/No |
| - Open text field for reporting the organ/limb | Open text field |
| - What was the age at surgery or year of surgery? | Open text field |

**Supplementary Table S2.** Other questionnaires addressing long-term morbidity in childhood cancer survivors: the Childhood Cancer Survivor Study questionnaire.

| **Childhood Cancer Survivor Study questionnaire** |  |
| --- | --- |
| **Question** | **Answer options** |
| **7. We would like to know all of the drugs and medications that you have taken during THE LAST 2 YEARS, THAT IS SINCE THE DATE EXACTLY 2 YEARS AGO. We are interested in only those tablets, pills, syrups, injections, patches and creams that were prescribed by a doctor, and which you took regularly for MORE THAN ONE MONTH, or for a total of 30 DAYS OR MORE IN A YEAR.** |  |
| 7a. Antibiotics  such as amoxycillin, penicillin, erythromycin, cephalexin or others | Yes/No/Not sure |
| Names of drugs | Open text field |
| 7b. Testosterone (male hormones)  such as Sustanon or others | Yes/No/Not sure |
| Names of drugs | Open text field |
| 7c. Thyroid medications  such as Thyroxine or others | Yes/No/Not sure |
| Names of drugs | Open text field |
| 7d. Other medications to replace body hormones  such as growth hormone, steroid hormones (hydrocortisone), DDAVP or others | Yes/No/Not sure |
| Names of drugs | Open text field |
| 7e. Medication for Diabetes  such as insulin, metformin (Glucophage), glibenclamide, gliclazide or others | Yes/No/Not sure |
| Names of drugs | Open text field |
| 7f. Muscle relaxants such as baclofen (Lioresal), dantrolene (Dantrium) or others | Yes/No/Not sure |
| Names of drugs | Open text field |
| 7g. Prescribed pain killers such as Solpadol, Tylex, diclofenac, naproxen, dihydrocodeine, morphine or others | Yes/No/Not sure |
| Names of drugs | Open text field |
| 7h. Prescribed nutritional supplements  such as iron tablets, magnesium, potassium, sodium bicarbonate, vitamin D or others | Yes/No/Not sure |
| Names of drugs | Open text field |
| 7i. Anti-epileptic (anti-seizure) drugs  such as phenytoin (Epanutin), carbamazepine (Tegretol), sodium valproate (Epilim), lamotrigine (Lamictal), ethosuximide (Zarontin), phenobarbitone or others | Yes/No/Not sure |
| Names of drugs | Open text field |
| 7j. Drugs for high blood pressure or for your heart  such as atenolol, captopril, enalapril, digoxin, frusemide or others | Yes/No/Not sure |
| Names of drugs | Open text field |
| 7k. Prescribed antacids (for excess stomach acid or ulcers)  such as cimetidine (Tagamet), ranitidine (Zantac),omeprazole (Losec) or others | Yes/No/Not sure |
| Names of drugs | Open text field |
| 7l. Chemotherapy or Immune suppressants  such as prednisolone, azathioprine, cyclosporin or others | Yes/No/Not sure |
| Names of drugs | Open text field |
| 7m. Antidepressants or other prescribed drugs for depression or other mood disorders  such as dothiepin (Prothiaden), amitriptyline, fluoxetine (Prozac), lithium or others | Yes/No/Not sure |
| Names of drugs | Open text field |
| 7n. Other prescribed drugs | Yes/No/Not sure |
| Names of drugs | Open text field |
| **The questions in this section ask about medical conditions you might have had which were confirmed by a doctor, nurse or other medical professional.**  **Has a doctor, nurse or other medical professional ever confirmed you have, or have had:** |  |
| **8. Brain and nervous system** |  |
| 8a. Cerebral palsy? |  |
| Yes/No/Not sure | Yes/No/Not sure |
| Please give age when it started (in years) | Open text field |
| 8b. Paralysis of any kind? |  |
| Yes/No/Not sure | Yes/No/Not sure |
| Please describe: | Open text field |
| Please give age when it started (in years) | Open text field |
| 8c. Mental retardation? |  |
| Yes/No/Not sure | Yes/No/Not sure |
| Please give age when it started (in years) | Open text field |
| 8d. Epilepsy? |  |
| Yes/No/Not sure | Yes/No/Not sure |
| Please give age when it started (in years) | Open text field |
| 8e. Repeated seizures, fits, convulsions, or blackouts? |  |
| Yes/No/Not sure | Yes/No/Not sure |
| Please give age when it started (in years) | Open text field |
| 8f. Migraine? |  |
| Yes/No/Not sure | Yes/No/Not sure |
| Please give age when it started (in years) | Open text field |
| 8g. Other frequent headaches? |  |
| Yes/No/Not sure | Yes/No/Not sure |
| Please give age when it started (in years) | Open text field |
| 8h. Problems with balance, equilibrium or ability to reach for, or manipulate, objects? |  |
| Yes/No/Not sure | Yes/No/Not sure |
| Please give age when it started (in years) | Open text field |
| 8i. Tremors or problems with movements? |  |
| Yes/No/Not sure | Yes/No/Not sure |
| Please give age when it started (in years) | Open text field |
| 8j. Weakness or inability to move your arm(s)? |  |
| Yes/No/Not sure | Yes/No/Not sure |
| Please give age when it started (in years) | Open text field |
| 8k. Weakness or inability to move your leg(s)? |  |
| Yes/No/Not sure | Yes/No/Not sure |
| Please give age when it started (in years) | Open text field |
| Yes/No/Not sure | Yes/No/Not sure |
| Please give age when it started (in years) | Open text field |
| 8l. Decreased sense of touch or feeling in your hands, fingers, arms or legs? |  |
| Yes/No/Not sure | Yes/No/Not sure |
| Please give age when it started (in years) | Open text field |
| 8m. Prolonged pain or abnormal sensation in arms, legs or back? |  |
| Yes/No/Not sure | Yes/No/Not sure |
| Please give age when it started (in years) | Open text field |
| 8n. Problems chewing or swallowing solids or liquids? |  |
| Yes/No/Not sure | Yes/No/Not sure |
| Please give age when it started (in years) | Open text field |
| 8o. Any other brain or nervous system problems? |  |
| Yes/No/Not sure | Yes/No/Not sure |
| Please describe: | Open text field |
| Please give age when it started (in years) | Open text field |
| **9. Kidneys, bladder and other urinary tract conditions** |  |
| 9a. Kidney stones? |  |
| Yes/No/Not sure | Yes/No/Not sure |
| Please give age when it started (in years) | Open text field |
| 9b. REPEATED kidney infections? |  |
| Yes/No/Not sure | Yes/No/Not sure |
| Please give age when it started (in years) | Open text field |
| 9c. REPEATED bladder infections? |  |
| Yes/No/Not sure | Yes/No/Not sure |
| Please give age when it started (in years) | Open text field |
| 9d. Dialysis? |  |
| Yes/No/Not sure | Yes/No/Not sure |
| Please give age when it started (in years) | Open text field |
| 9e. Any other kind of kidney or urinary tract disorder? |  |
| Yes/No/Not sure | Yes/No/Not sure |
| Please describe: | Open text field |
| Please give age when it started (in years) | Open text field |
| **10. Digestive system** |  |
| 10a. Gallstones? |  |
| Yes/No/Not sure | Yes/No/Not sure |
| Please give age when it started (in years) | Open text field |
| 10b. Any other gallbladder trouble? |  |
| Yes/No/Not sure | Yes/No/Not sure |
| Please describe: | Open text field |
| Please give age when it started (in years) | Open text field |
| 10c. Cirrhosis of the liver? |  |
| Yes/No/Not sure | Yes/No/Not sure |
| Please give age when it started (in years) | Open text field |
| 10d. Hepatitis? |  |
| Yes/No/Not sure | Yes/No/Not sure |
| Please give age when it started (in years) | Open text field |
| 10e. Jaundice? |  |
| Yes/No/Not sure | Yes/No/Not sure |
| Please give age when it started (in years) | Open text field |
| 10f. Any other liver trouble? |  |
| Yes/No/Not sure | Yes/No/Not sure |
| Please describe: | Open text field |
| Please give age when it started (in years) | Open text field |
| 10g. An ulcer? |  |
| Yes/No/Not sure | Yes/No/Not sure |
| Please give age when it started (in years) | Open text field |
| 10h. Any disease of the oesophagus? |  |
| Yes/No/Not sure | Yes/No/Not sure |
| Please describe: | Open text field |
| Please give age when it started (in years) | Open text field |
| 10i. FREQUENT indigestion? |  |
| Yes/No/Not sure | Yes/No/Not sure |
| Please give age when it started (in years) | Open text field |
| 10j. FREQUENT heartburn? |  |
| Yes/No/Not sure | Yes/No/Not sure |
| IF yes: did you take medication for it for more than once a month? | Yes/No/Not sure |
| Please give age when it started (in years) | Open text field |
| 10k. Any other stomach trouble? |  |
| Yes/No/Not sure | Yes/No/Not sure |
| Please describe: | Open text field |
| Please give age when it started (in years) | Open text field |
| 10l. Intestinal polyps? |  |
| Yes/No/Not sure | Yes/No/Not sure |
| Please give age when it started (in years) | Open text field |
| 10m. Diverticular disease? |  |
| Yes/No/Not sure | Yes/No/Not sure |
| Please give age when it started (in years) | Open text field |
| 10n. Colitis? |  |
| Yes/No/Not sure | Yes/No/Not sure |
| Please give age when it started (in years) | Open text field |
| 10o. FREQUENT constipation? |  |
| Yes/No/Not sure | Yes/No/Not sure |
| Please give age when it started (in years) | Open text field |
| 10p. Chronic diarrhoea? |  |
| Yes/No/Not sure | Yes/No/Not sure |
| Please give age when it started (in years) | Open text field |
| 10q. Rectal or anal fistula? |  |
| Yes/No/Not sure | Yes/No/Not sure |
| Please give age when it started (in years) | Open text field |
| 10r. Rectal or anal stricture (narrowing or scarring)? |  |
| Yes/No/Not sure | Yes/No/Not sure |
| Please give age when it started (in years) | Open text field |
| 10s. Any other digestive problems? |  |
| Yes/No/Not sure | Yes/No/Not sure |
| Please describe: | Open text field |
| Please give age when it started (in years) | Open text field |
| **11. Hormonal system** |  |
| 11a. An overactive thyroid gland (hyperthyroid)? |  |
| Yes/No/Not sure | Yes/No/Not sure |
| Please give age when it started (in years) | Open text field |
| 11b. An underactive thyroid gland (hypothyroid)? |  |
| Yes/No/Not sure | Yes/No/Not sure |
| Please give age when it started (in years) | Open text field |
| 11c. Thyroid nodules? |  |
| Yes/No/Not sure | Yes/No/Not sure |
| Please give age when it started (in years) | Open text field |
| 11d. Other thyroid enlargements? |  |
| Yes/No/Not sure | Yes/No/Not sure |
| Please give age when it started (in years) | Open text field |
| 11d. Diabetes - controlled with diet? ^A^ |  |
| Yes/No/Not sure | Yes/No/Not sure |
| Please give age when it started (in years) | Open text field |
| 11e. Diabetes - controlled with pills or tablets? |  |
| Yes/No/Not sure | Yes/No/Not sure |
| Please give age when it started (in years) | Open text field |
| 11f. Diabetes - controlled with insulin injections? |  |
| Yes/No/Not sure | Yes/No/Not sure |
| Please give age when it started (in years) | Open text field |
| 11g. Lack of growth hormone? |  |
| Yes/No/Not sure | Yes/No/Not sure |
| Please give age when it started (in years) | Open text field |
| 11h. Have you ever received injections of growth hormone? |  |
| Yes/No/Not sure | Yes/No/Not sure |
| Please give age when it started (in years) | Open text field |
| 11i. Osteoporosis, brittle, weak or fragile bones? |  |
| Yes/No/Not sure | Yes/No/Not sure |
| Please give age when it started (in years) | Open text field |
| 11j. Did you need medication to go into puberty? |  |
| Yes/No/Not sure | Yes/No/Not sure |
| Please give age when it started (in years) | Open text field |
| 11k. Any other hormonal problems? |  |
| Yes/No/Not sure | Yes/No/Not sure |
| Please describe: | Open text field |
| Please give age when it started (in years) | Open text field |
| **12. Lungs and breathing** |  |
| 12a. Bronchitis? |  |
| Yes/No/Not sure | Yes/No/Not sure |
| Please give age when it started (in years) | Open text field |
| 12b. Hay fever? |  |
| Yes/No/Not sure | Yes/No/Not sure |
| Please give age when it started (in years) | Open text field |
| 12c. Recurrent sinus infections? |  |
| Yes/No/Not sure | Yes/No/Not sure |
| Please give age when it started (in years) | Open text field |
| 12d. Tonsillitis or enlargement of the tonsils or adenoids? |  |
| Yes/No/Not sure | Yes/No/Not sure |
| Please give age when it started (in years) | Open text field |
| 12e. Pleurisy (inflammation of the lining of the lung)? |  |
| Yes/No/Not sure | Yes/No/Not sure |
| Please give age when it started (in years) | Open text field |
| 12f. Asthma? |  |
| Yes/No/Not sure | Yes/No/Not sure |
| Please give age when it started (in years) | Open text field |
| 12g. Abnormal chest wall? |  |
| Yes/No/Not sure | Yes/No/Not sure |
| Please give age when it started (in years) | Open text field |
| 12h. Chronic cough or shortness of breath for more than a month? |  |
| Yes/No/Not sure | Yes/No/Not sure |
| Please give age when it started (in years) | Open text field |
| 12i. Have you ever had a need for extra oxygen? |  |
| Yes/No/Not sure | Yes/No/Not sure |
| IF yes: are you currently using extra oxygen? | Yes/No/Not sure |
| Please give age when it started (in years) | Open text field |
| 12j. Pneumonia 3 or more times in the past 2 years? |  |
| Yes/No/Not sure | Yes/No/Not sure |
| Please give age when it started (in years) | Open text field |
| 12k. Emphysema? |  |
| Yes/No/Not sure | Yes/No/Not sure |
| Please give age when it started (in years) | Open text field |
| 12l. Lung fibrosis or “scarring” of the lung? |  |
| Yes/No/Not sure | Yes/No/Not sure |
| Please give age when it started (in years) | Open text field |
| 12m. Any other breathing or lung problems? |  |
| Yes/No/Not sure | Yes/No/Not sure |
| If yes: please describe | Open text field |
| Please give age when it started (in years) | Open text field |
| **13. Heart and circulatory system** |  |
| 13a. Rheumatic heart disease? |  |
| Yes/No/Not sure | Yes/No/Not sure |
| Please give age when it started (in years) | Open text field |
| 13b. Hardening of the arteries or arteriosclerosis? |  |
| Yes/No/Not sure | Yes/No/Not sure |
| Please give age when it started (in years) | Open text field |
| 13c. Irregular heart beat or palpitations, (Arrhythmia) requiring medication or follow-up by a doctor? |  |
| Yes/No/Not sure | Yes/No/Not sure |
| Please give age when it started (in years) | Open text field |
| 13d. Congestive heart failure or cardiomyopathy (weak heart muscle)? |  |
| Yes/No/Not sure | Yes/No/Not sure |
| Please give age when it started (in years) | Open text field |
| 13e. A myocardial infarction (heart attack)? |  |
| Yes/No/Not sure | Yes/No/Not sure |
| Please give age when it started (in years) | Open text field |
| 13f. Coronary heart disease? |  |
| Yes/No/Not sure | Yes/No/Not sure |
| Please give age when it started (in years) | Open text field |
| 13g. A heart murmur? |  |
| Yes/No/Not sure | Yes/No/Not sure |
| Please give age when it started (in years) | Open text field |
| 13h. Hypertension (high blood pressure) not requiring medication? |  |
| Yes/No/Not sure | Yes/No/Not sure |
| Please give age when it started (in years) | Open text field |
| 13i. Hypertension (high blood pressure) requiring medication? |  |
| Yes/No/Not sure | Yes/No/Not sure |
| Please give age when it started (in years) | Open text field |
| 13j. A stroke or a cerebrovascular accident? |  |
| Yes/No/Not sure | Yes/No/Not sure |
| Please give age when it started (in years) | Open text field |
| 13k. Angina pectoris (chest pains due to lack of oxygen to heart requiring medication such as Glyceryl  Trinitrate sometimes known as GTN)? |  |
| Yes/No/Not sure | Yes/No/Not sure |
| Please give age when it started (in years) | Open text field |
| 13l. Pericarditis or fluid around the heart? |  |
| Yes/No/Not sure | Yes/No/Not sure |
| Please give age when it started (in years) | Open text field |
| 13m. Pericardial constriction (scarring or tightness of the sac around the heart)? |  |
| Yes/No/Not sure | Yes/No/Not sure |
| Please give age when it started (in years) | Open text field |
| 13n. Stiff or leaking heart valves? |  |
| Yes/No/Not sure | Yes/No/Not sure |
| Please give age when it started (in years) | Open text field |
| 13o. Heart catheterisation? |  |
| Yes/No/Not sure | Yes/No/Not sure |
| Please give age when it started (in years) | Open text field |
| 13p. Biopsy of the heart muscle? |  |
| Yes/No/Not sure | Yes/No/Not sure |
| Please give age when it started (in years) | Open text field |
| 13q. Blood clot in head, lung, arm, leg or pelvis? |  |
| Yes/No/Not sure | Yes/No/Not sure |
| Please give age when it started (in years) | Open text field |
| 13r. Does exercise cause severe chest pain, shortness of breath, or irregular heart beat? |  |
| Yes/No/Not sure | Yes/No/Not sure |
| Please give age when it started (in years) | Open text field |
| 13s. Have you seen a cardiologist (heart specialist)? |  |
| Yes/No/Not sure | Yes/No/Not sure |
| Please give age when it started (in years) | Open text field |
| 13t. Has anyone in your immediate family (biological mother, father, brothers, sisters) had a heart attack  before the age of 55? |  |
| Yes/No/Not sure | Yes/No/Not sure |
| Please give age when it started (in years) | Open text field |
| 13u. Any other heart or circulatory problems? |  |
| Yes/No/Not sure | Yes/No/Not sure |
| If yes: please describe | Open text field |
| Please give age when it started (in years) | Open text field |
| **14. Hearing, vision, speech and taste** |  |
| 14a. Hearing loss requiring a hearing aid? |  |
| Yes/No/Not sure | Yes/No/Not sure |
| Please give age when it started (in years) | Open text field |
| 14b. Deafness in one or both ears not completely corrected by a hearing aid? |  |
| Yes/No/Not sure | Yes/No/Not sure |
| Please give age when it started (in years) | Open text field |
| 14c. Complete deafness in either ear? |  |
| Yes/No/Not sure | Yes/No/Not sure |
| Please give age when it started (in years) | Open text field |
| 14d. Tinnitus or ringing in the ears? |  |
| Yes/No/Not sure | Yes/No/Not sure |
| Please give age when it started (in years) | Open text field |
| 14e. Persistent dizziness or vertigo? |  |
| Yes/No/Not sure | Yes/No/Not sure |
| Please give age when it started (in years) | Open text field |
| 14f. Problems hearing sounds, words, or language in crowds? |  |
| Yes/No/Not sure | Yes/No/Not sure |
| Please give age when it started (in years) | Open text field |
| 14g. Any other hearing problems? |  |
| Yes/No/Not sure | Yes/No/Not sure |
| If yes: please describe | Open text field |
| Please give age when it started (in years) | Open text field |
| 14h. Registered as blind? |  |
| Yes/No/Not sure | Yes/No/Not sure |
| Please give age when it started (in years) | Open text field |
| 14i. Cataracts? |  |
| Yes/No/Not sure | Yes/No/Not sure |
| Please give age when it started (in years) | Open text field |
| 14j. Glaucoma (excess pressure in the eyeball)? |  |
| Yes/No/Not sure | Yes/No/Not sure |
| Please give age when it started (in years) | Open text field |
| 14k. Problems with double vision? |  |
| Yes/No/Not sure | Yes/No/Not sure |
| Please give age when it started (in years) | Open text field |
| 14l. A detached retina or any other condition of the retina? |  |
| Yes/No/Not sure | Yes/No/Not sure |
| If yes: please describe | Open text field |
| Please give age when it started (in years) | Open text field |
| 14m. Any other trouble seeing with one or both eyes even when wearing glasses? |  |
| Yes/No/Not sure | Yes/No/Not sure |
| Please give age when it started (in years) | Open text field |
| 14n. Very dry eyes requiring eye drops or ointment? |  |
| Yes/No/Not sure | Yes/No/Not sure |
| Please give age when it started (in years) | Open text field |
| 14o. Short-sightedness (Myopia) |  |
| Yes/No/Not sure | Yes/No/Not sure |
| Please give age when it started (in years) | Open text field |
| 14p. Long-sightedness (Hypermetropia) |  |
| Yes/No/Not sure | Yes/No/Not sure |
| Please give age when it started (in years) | Open text field |
| 14q. Any other eye problems? |  |
| Yes/No/Not sure | Yes/No/Not sure |
| If yes: please describe | Open text field |
| Please give age when it started (in years) | Open text field |
| 14r. Stammering or stuttering? |  |
| Yes/No/Not sure | Yes/No/Not sure |
| Please give age when it started (in years) | Open text field |
| 14s. Any other speech defects? |  |
| Yes/No/Not sure | Yes/No/Not sure |
| If yes: please describe | Open text field |
| Please give age when it started (in years) | Open text field |
| 14t. Abnormal sense of taste? |  |
| Yes/No/Not sure | Yes/No/Not sure |
| Please give age when it started (in years) | Open text field |
| 14u. Loss of taste or smell which lasted for 3 months or more? |  |
| Yes/No/Not sure | Yes/No/Not sure |
| If yes: please describe | Open text field |
| Please give age when it started (in years) | Open text field |
| **15. It is important to know whether some forms of medical treatment in childhood affect a man’s potential to father a child.** |  |
| 15a. Has a doctor ever told you that you might have problems fathering a child? |  |
| Yes/No/Not sure | Yes/No/Not sure |
| Please give age when it started (in years) | Open text field |
| 15b. Have you ever had medical tests (such as a blood test, ultrasound or sperm count) to see whether or  not you might have problems fathering a child? |  |
| Yes/No/Not sure | Yes/No/Not sure |
| Please give age when it started (in years) | Open text field |
| 15c. Have you ever been told that you have a low sperm count? |  |
| **16. please indicate if you have ever had any of the following types of surgery and give your approximate age when you first had each type of operation. Please also indicate which types of surgery you never had by ticking the no box.** |  |
| 16a. Amputation of an arm, leg, hand, foot, finger or toe? |  |
| Yes/No/Not sure | Yes/No/Not sure |
| If yes: please describe | Open text field |
| Please give age when it started (in years) | Open text field |
| 16b. Scoliosis surgery (insertion of rods or other methods to straighten the spine)? |  |
| Yes/No/Not sure | Yes/No/Not sure |
| Please give age when it started (in years) | Open text field |
| 16c. Other surgery of your spinal cord or spine? |  |
| Yes/No/Not sure | Yes/No/Not sure |
| If yes: please describe | Open text field |
| Please give age when it started (in years) | Open text field |
| 16d. Leg lengthening or shortening operations? |  |
| Yes/No/Not sure | Yes/No/Not sure |
| Please give age when it started (in years) | Open text field |
| 16e. Joint replacement operations? |  |
| Yes/No/Not sure | Yes/No/Not sure |
| If yes: please describe | Open text field |
| Please give age when it started (in years) | Open text field |
| 16f. Other bone surgery? |  |
| Yes/No/Not sure | Yes/No/Not sure |
| Please give age when it started (in years) | Open text field |
| 16g. Coronary artery bypass surgery? |  |
| Yes/No/Not sure | Yes/No/Not sure |
| Please give age when it started (in years) | Open text field |
| 16h. Pericardiectomy (stripping of the sac around the heart)? |  |
| Yes/No/Not sure | Yes/No/Not sure |
| Please give age when it started (in years) | Open text field |
| 16i. Angioplasty (enlarging a heart vessel using a balloon)? |  |
| Yes/No/Not sure | Yes/No/Not sure |
| Please give age when it started (in years) | Open text field |
| 16j. Other heart surgery? |  |
| Yes/No/Not sure | Yes/No/Not sure |
| If yes: please describe | Open text field |
| Please give age when it started (in years) | Open text field |
| 16k. Surgery for intestinal obstruction (blocked intestines)? |  |
| Yes/No/Not sure | Yes/No/Not sure |
| Please give age when it started (in years) | Open text field |
| 16l. Colostomy or ileostomy (stool going into a bag)? |  |
| Yes/No/Not sure | Yes/No/Not sure |
| Please give age when it started (in years) | Open text field |
| 16m. Reconnection after a colostomy or ileostomy? |  |
| Yes/No/Not sure | Yes/No/Not sure |
| Please give age when it started (in years) | Open text field |
| 16n. Surgery to remove a blood clot in an artery or vein? |  |
| Yes/No/Not sure | Yes/No/Not sure |
| If yes: please describe | Open text field |
| Please give age when it started (in years) | Open text field |
| 16o. Removal of the thyroid gland in your neck? |  |
| Yes/No/Not sure | Yes/No/Not sure |
| Please give age when it started (in years) | Open text field |
| 16p. Removal of your spleen? |  |
| Yes/No/Not sure | Yes/No/Not sure |
| Please give age when it started (in years) | Open text field |
| 16q. Ventriculoperitoneal shunt (tube from the brain to the abdomen, under the skin, which removes excess spinal fluid)? |  |
| Yes/No/Not sure | Yes/No/Not sure |
| Please give age when it started (in years) | Open text field |
| 16r. Breast surgery for removal or biopsy of a suspicious lump? |  |
| Yes/No/Not sure | Yes/No/Not sure |
| Please give age when it started (in years) | Open text field |
| 16s. A bronchoscopy since your therapy stopped? |  |
| Yes/No/Not sure | Yes/No/Not sure |
| Please give age when it started (in years) | Open text field |
| 16t. Other lung surgery? |  |
| Yes/No/Not sure | Yes/No/Not sure |
| If yes: please describe | Open text field |
| Please give age when it started (in years) | Open text field |
| 16u. A liver biopsy since your therapy stopped? |  |
| Yes/No/Not sure | Yes/No/Not sure |
| Please give age when it started (in years) | Open text field |
| 16v. Reconstructive surgery (surgery to repair damage due to an accident or medical therapy or other surgery)? |  |
| Yes/No/Not sure | Yes/No/Not sure |
| If yes: please describe | Open text field |
| Please give age when it started (in years) | Open text field |
| 16w. A heart transplant? |  |
| Yes/No/Not sure | Yes/No/Not sure |
| Please give age when it started (in years) | Open text field |
| 16x. A lung transplant? |  |
| Yes/No/Not sure | Yes/No/Not sure |
| Please give age when it started (in years) | Open text field |
| 16y. A kidney transplant? |  |
| Yes/No/Not sure | Yes/No/Not sure |
| Please give age when it started (in years) | Open text field |
| 16z. A bone marrow transplant? |  |
| Yes/No/Not sure | Yes/No/Not sure |
| Please give age when it started (in years) | Open text field |
| 17a. Other organ transplant? |  |
| Yes/No/Not sure | Yes/No/Not sure |
| If yes: please describe | Open text field |
| Please give age when it started (in years) | Open text field |
| 17b. Cataract surgery? |  |
| Yes/No/Not sure | Yes/No/Not sure |
| Please give age when it started (in years) | Open text field |
| 17c. Sinus surgery? |  |
| Yes/No/Not sure | Yes/No/Not sure |
| Please give age when it started (in years) | Open text field |
| 17d. Surgery on your jaw? |  |
| Yes/No/Not sure | Yes/No/Not sure |
| Please give age when it started (in years) | Open text field |
| 17e. Any other surgery? |  |
| Yes/No/Not sure | Yes/No/Not sure |
| If yes: please describe | Open text field |
| Please give age when it started (in years) | Open text field |
| **18a. Did your childhood cancer, leukaemia, tumour or similar illness ever come back (recur or relapse)**  **after it was first treated?** |  |
| Yes/No | Yes/No |
| If yes, please give details of the first time it came back after the first treatment |  |
| - Date doctor confirmed illness has come back | DD/MM/YYYY |
| - Doctor’s name | Open text field |
| - Hospital name | Open text field |
| - Hospital address | Open text field |
| **18b. Apart from your original childhood cancer, leukaemia, tumour or similar illness, have you ever**  **been diagnosed with any OTHER cancer, leukaemia or tumour?** |  |
| Yes/No | Yes/No |
| If yes, please give details of the first time it came back after the first treatment |  |
| - Date doctor confirmed illness has come back | Open text field |
| - Illness diagnosed | DD/MM/YYYY |
| - Doctor’s name | Open text field |
| - Hospital name | Open text field |
| - Hospital address | Open text field |
|  |  |

^A^ Question no. 11.b was numbered twice in the original Childhood Cancer Survivor Study questionnaire.

**Supplementary Table S3.** Other questionnaires addressing long-term morbidity in childhood cancer survivors: the British Childhood Cancer Survivor Study questionnaire.

| **British Childhood Cancer Survivor Study questionnaire** |  |
| --- | --- |
| **Question** | **Answer options** |
| **7. We would like to know all of the drugs and medications that you have taken during THE LAST 2 YEARS, THAT IS SINCE THE DATE EXACTLY 2 YEARS AGO. We are interested in only those tablets, pills, syrups, injections, patches and creams that were prescribed by a doctor, and which you took regularly for MORE THAN ONE MONTH, or for a total of 30 DAYS OR MORE IN A YEAR.** |  |
| 7a. Antibiotics  such as amoxycillin, penicillin, erythromycin, cephalexin or others | Yes/No/Not sure |
| Names of drugs | Open tekst field |
| 7b. Testosterone (male hormones)  such as Sustanon or others | Yes/No/Not sure |
| Names of drugs | Open tekst field |
| 7c. Thyroid medications  such as Thyroxine or others | Yes/No/Not sure |
| Names of drugs | Open tekst field |
| 7d. Other medications to replace body hormones  such as growth hormone, steroid hormones (hydrocortisone), DDAVP or others | Yes/No/Not sure |
| Names of drugs | Open tekst field |
| 7e. Medication for Diabetes  such as insulin, metformin (Glucophage), glibenclamide, gliclazide or others | Yes/No/Not sure |
| Names of drugs | Open tekst field |
| 7f. Muscle relaxants such as baclofen (Lioresal), dantrolene (Dantrium) or others | Yes/No/Not sure |
| Names of drugs | Open tekst field |
| 7g. Prescribed pain killers such as Solpadol, Tylex, diclofenac, naproxen, dihydrocodeine, morphine or others | Yes/No/Not sure |
| Names of drugs | Open tekst field |
| 7h. Prescribed nutritional supplements  such as iron tablets, magnesium, potassium, sodium bicarbonate, vitamin D or others | Yes/No/Not sure |
| Names of drugs | Open tekst field |
| 7i. Anti-epileptic (anti-seizure) drugs  such as phenytoin (Epanutin), carbamazepine (Tegretol), sodium valproate (Epilim), lamotrigine (Lamictal), ethosuximide (Zarontin), phenobarbitone or others | Yes/No/Not sure |
| Names of drugs | Open tekst field |
| 7j. Drugs for high blood pressure or for your heart  such as atenolol, captopril, enalapril, digoxin, furosemide or others | Yes/No/Not sure |
| Names of drugs | Open tekst field |
| 7k. Prescribed antacids (for excess stomach acid or ulcers)  such as cimetidine (Tagamet), ranitidine (Zantac),omeprazole (Losec) or others | Yes/No/Not sure |
| Names of drugs | Open tekst field |
| 7l. Chemotherapy or Immune suppressants  such as prednisolone, azathioprine, cyclosporin or others | Yes/No/Not sure |
| Names of drugs | Open tekst field |
| 7m. Antidepressants or other prescribed drugs for depression or other mood disorders  such as dothiepin (Prothiaden), amitriptyline, fluoxetine (Prozac), lithium or others | Yes/No/Not sure |
| Names of drugs | Open tekst field |
| 7n. Other prescribed drugs | Yes/No/Not sure |
| Names of drugs | Open tekst field |
| **The questions in this section ask about medical conditions you might have had which were confirmed by a doctor, nurse or other medical professional.**  **Has a doctor, nurse or other medical professional ever confirmed you have, or have had:** |  |
| **Brain and nervous system** |  |
| 8a. Cerebral palsy? |  |
| Yes/No/Not sure | Yes/No/Not sure |
| Please give age when it started (in years) | Open text field |
| 8b. Paralysis of any kind? |  |
| Yes/No/Not sure | Yes/No/Not sure |
| Please describe: | Open text field |
| Please give age when it started (in years) | Open text field |
| 8c. Mental retardation? |  |
| Yes/No/Not sure | Yes/No/Not sure |
| Please give age when it started (in years) | Open text field |
| 8d. Epilepsy? |  |
| Yes/No/Not sure | Yes/No/Not sure |
| Please give age when it started (in years) | Open text field |
| 8e. Repeated seizures, fits, convulsions, or blackouts? |  |
| Yes/No/Not sure | Yes/No/Not sure |
| Please give age when it started (in years) | Open text field |
| 8f. Migraine? |  |
| Yes/No/Not sure | Yes/No/Not sure |
| Please give age when it started (in years) | Open text field |
| 8g. Other frequent headaches? |  |
| Yes/No/Not sure | Yes/No/Not sure |
| Please give age when it started (in years) | Open text field |
| 8h. Problems with balance, equilibrium or ability to reach for, or manipulate, objects? |  |
| Yes/No/Not sure | Yes/No/Not sure |
| Please give age when it started (in years) | Open text field |
| 8i. Tremors or problems with movements? |  |
| Yes/No/Not sure | Yes/No/Not sure |
| Please give age when it started (in years) | Open text field |
| 8j. Weakness or inability to move your arm(s)? |  |
| Yes/No/Not sure | Yes/No/Not sure |
| Please give age when it started (in years) | Open text field |
| 8k. Weakness or inability to move your leg(s)? |  |
| Yes/No/Not sure | Yes/No/Not sure |
| Please give age when it started (in years) | Open text field |
| Yes/No/Not sure | Yes/No/Not sure |
| Please give age when it started (in years) | Open text field |
| 8l. Decreased sense of touch or feeling in your hands, fingers, arms or legs? |  |
| Yes/No/Not sure | Yes/No/Not sure |
| Please give age when it started (in years) | Open text field |
| 8m. Prolonged pain or abnormal sensation in arms, legs or back? |  |
| Yes/No/Not sure | Yes/No/Not sure |
| Please give age when it started (in years) | Open text field |
| 8n. Problems chewing or swallowing solids or liquids? |  |
| Yes/No/Not sure | Yes/No/Not sure |
| Please give age when it started (in years) | Open text field |
| 8o. Any other brain or nervous system problems? |  |
| Yes/No/Not sure | Yes/No/Not sure |
| Please describe: | Open text field |
| Please give age when it started (in years) | Open text field |
| **Kidneys, bladder and other urinary tract conditions** |  |
| 9a. Kidney stones? |  |
| Yes/No/Not sure | Yes/No/Not sure |
| Please give age when it started (in years) | Open text field |
| 9b. REPEATED kidney infections? |  |
| Yes/No/Not sure | Yes/No/Not sure |
| Please give age when it started (in years) | Open text field |
| 9c. REPEATED bladder infections? |  |
| Yes/No/Not sure | Yes/No/Not sure |
| Please give age when it started (in years) | Open text field |
| 9d. Dialysis? |  |
| Yes/No/Not sure | Yes/No/Not sure |
| Please give age when it started (in years) | Open text field |
| 9e. Any other kind of kidney or urinary tract disorder? |  |
| Yes/No/Not sure | Yes/No/Not sure |
| Please describe: | Open text field |
| Please give age when it started (in years) | Open text field |
| **Digestive system** |  |
| 10a. Gallstones? |  |
| Yes/No/Not sure | Yes/No/Not sure |
| Please give age when it started (in years) | Open text field |
| 10b. Any other gallbladder trouble? |  |
| Yes/No/Not sure | Yes/No/Not sure |
| Please describe: | Open text field |
| Please give age when it started (in years) | Open text field |
| 10c. Cirrhosis of the liver? |  |
| Yes/No/Not sure | Yes/No/Not sure |
| Please give age when it started (in years) | Open text field |
| 10d. Hepatitis? |  |
| Yes/No/Not sure | Yes/No/Not sure |
| Please give age when it started (in years) | Open text field |
| 10e. Jaundice? |  |
| Yes/No/Not sure | Yes/No/Not sure |
| Please give age when it started (in years) | Open text field |
| 10f. Any other liver trouble? |  |
| Yes/No/Not sure | Yes/No/Not sure |
| Please describe: | Open text field |
| Please give age when it started (in years) | Open text field |
| 10g. An ulcer? |  |
| Yes/No/Not sure | Yes/No/Not sure |
| Please give age when it started (in years) | Open text field |
| 10h. Any disease of the oesophagus? |  |
| Yes/No/Not sure | Yes/No/Not sure |
| Please describe: | Open text field |
| Please give age when it started (in years) | Open text field |
| 10i. FREQUENT indigestion? |  |
| Yes/No/Not sure | Yes/No/Not sure |
| Please give age when it started (in years) | Open text field |
| 10j. FREQUENT heartburn? |  |
| Yes/No/Not sure | Yes/No/Not sure |
| IF yes: did you take medication for it for more than once a month? | Yes/No/Not sure |
| Please give age when it started (in years) | Open text field |
| 10k. Any other stomach trouble? |  |
| Yes/No/Not sure | Yes/No/Not sure |
| Please describe: | Open text field |
| Please give age when it started (in years) | Open text field |
| 10l. Intestinal polyps? |  |
| Yes/No/Not sure | Yes/No/Not sure |
| Please give age when it started (in years) | Open text field |
| 10m. Diverticular disease? |  |
| Yes/No/Not sure | Yes/No/Not sure |
| Please give age when it started (in years) | Open text field |
| 10n. Colitis? |  |
| Yes/No/Not sure | Yes/No/Not sure |
| Please give age when it started (in years) | Open text field |
| 10o. FREQUENT constipation? |  |
| Yes/No/Not sure | Yes/No/Not sure |
| Please give age when it started (in years) | Open text field |
| 10p. Chronic diarrhoea? |  |
| Yes/No/Not sure | Yes/No/Not sure |
| Please give age when it started (in years) | Open text field |
| 10q. Rectal or anal fistula? |  |
| Yes/No/Not sure | Yes/No/Not sure |
| Please give age when it started (in years) | Open text field |
| 10r. Rectal or anal stricture (narrowing or scarring)? |  |
| Yes/No/Not sure | Yes/No/Not sure |
| Please give age when it started (in years) | Open text field |
| 10s. Any other digestive problems? |  |
| Yes/No/Not sure | Yes/No/Not sure |
| Please describe: | Open text field |
| Please give age when it started (in years) | Open text field |
| **Hormonal system** |  |
| 11a. An overactive thyroid gland (hyperthyroid)? |  |
| Yes/No/Not sure | Yes/No/Not sure |
| Please give age when it started (in years) | Open text field |
| 11b. An underactive thyroid gland (hypothyroid)? |  |
| Yes/No/Not sure | Yes/No/Not sure |
| Please give age when it started (in years) | Open text field |
| 11c. Thyroid nodules? |  |
| Yes/No/Not sure | Yes/No/Not sure |
| Please give age when it started (in years) | Open text field |
| 11d. Other thyroid enlargements? |  |
| Yes/No/Not sure | Yes/No/Not sure |
| Please give age when it started (in years) | Open text field |
| 11d. Diabetes - controlled with diet? ^A^ |  |
| Yes/No/Not sure | Yes/No/Not sure |
| Please give age when it started (in years) | Open text field |
| 11e. Diabetes - controlled with pills or tablets? |  |
| Yes/No/Not sure | Yes/No/Not sure |
| Please give age when it started (in years) | Open text field |
| 11f. Diabetes - controlled with insulin injections? |  |
| Yes/No/Not sure | Yes/No/Not sure |
| Please give age when it started (in years) | Open text field |
| 11g. Lack of growth hormone? |  |
| Yes/No/Not sure | Yes/No/Not sure |
| Please give age when it started (in years) | Open text field |
| 11h. Have you ever received injections of growth hormone? |  |
| Yes/No/Not sure | Yes/No/Not sure |
| Please give age when it started (in years) | Open text field |
| 11i. Osteoporosis, brittle, weak or fragile bones? |  |
| Yes/No/Not sure | Yes/No/Not sure |
| Please give age when it started (in years) | Open text field |
| 11j. Did you need medication to go into puberty? |  |
| Yes/No/Not sure | Yes/No/Not sure |
| Please give age when it started (in years) | Open text field |
| 11k. Any other hormonal problems? |  |
| Yes/No/Not sure | Yes/No/Not sure |
| Please describe: | Open text field |
| Please give age when it started (in years) | Open text field |
| **Lungs and breathing** |  |
| 12a. Bronchitis? |  |
| Yes/No/Not sure | Yes/No/Not sure |
| Please give age when it started (in years) | Open text field |
| 12b. Hay fever? |  |
| Yes/No/Not sure | Yes/No/Not sure |
| Please give age when it started (in years) | Open text field |
| 12c. Recurrent sinus infections? |  |
| Yes/No/Not sure | Yes/No/Not sure |
| Please give age when it started (in years) | Open text field |
| 12d. Tonsillitis or enlargement of the tonsils or adenoids? |  |
| Yes/No/Not sure | Yes/No/Not sure |
| Please give age when it started (in years) | Open text field |
| 12e. Pleurisy (inflammation of the lining of the lung)? |  |
| Yes/No/Not sure | Yes/No/Not sure |
| Please give age when it started (in years) | Open text field |
| 12f. Asthma? |  |
| Yes/No/Not sure | Yes/No/Not sure |
| Please give age when it started (in years) | Open text field |
| 12g. Abnormal chest wall? |  |
| Yes/No/Not sure | Yes/No/Not sure |
| Please give age when it started (in years) | Open text field |
| 12h. Chronic cough or shortness of breath for more than a month? |  |
| Yes/No/Not sure | Yes/No/Not sure |
| Please give age when it started (in years) | Open text field |
| 12i. Have you ever had a need for extra oxygen? |  |
| Yes/No/Not sure | Yes/No/Not sure |
| IF yes: are you currently using extra oxygen? | Yes/No/Not sure |
| Please give age when it started (in years) | Open text field |
| 12j. Pneumonia 3 or more times in the past 2 years? |  |
| Yes/No/Not sure | Yes/No/Not sure |
| Please give age when it started (in years) | Open text field |
| 12k. Emphysema? |  |
| Yes/No/Not sure | Yes/No/Not sure |
| Please give age when it started (in years) | Open text field |
| 12l. Lung fibrosis or “scarring” of the lung? |  |
| Yes/No/Not sure | Yes/No/Not sure |
| Please give age when it started (in years) | Open text field |
| 12m. Any other breathing or lung problems? |  |
| Yes/No/Not sure | Yes/No/Not sure |
| If yes: please describe | Open text field |
| Please give age when it started (in years) | Open text field |
| **Heart and circulatory system** |  |
| 13a. Rheumatic heart disease? |  |
| Yes/No/Not sure | Yes/No/Not sure |
| Please give age when it started (in years) | Open text field |
| 13b. Hardening of the arteries or arteriosclerosis? |  |
| Yes/No/Not sure | Yes/No/Not sure |
| Please give age when it started (in years) | Open text field |
| 13c. Irregular heart beat or palpitations, (Arrhythmia) requiring medication or follow-up by a doctor? |  |
| Yes/No/Not sure | Yes/No/Not sure |
| Please give age when it started (in years) | Open text field |
| 13d. Congestive heart failure or cardiomyopathy (weak heart muscle)? |  |
| Yes/No/Not sure | Yes/No/Not sure |
| Please give age when it started (in years) | Open text field |
| 13e. A myocardial infarction (heart attack)? |  |
| Yes/No/Not sure | Yes/No/Not sure |
| Please give age when it started (in years) | Open text field |
| 13f. Coronary heart disease? |  |
| Yes/No/Not sure | Yes/No/Not sure |
| Please give age when it started (in years) | Open text field |
| 13g. A heart murmur? |  |
| Yes/No/Not sure | Yes/No/Not sure |
| Please give age when it started (in years) | Open text field |
| 13h. Hypertension (high blood pressure) not requiring medication? |  |
| Yes/No/Not sure | Yes/No/Not sure |
| Please give age when it started (in years) | Open text field |
| 13i. Hypertension (high blood pressure) requiring medication? |  |
| Yes/No/Not sure | Yes/No/Not sure |
| Please give age when it started (in years) | Open text field |
| 13j. A stroke or a cerebrovascular accident? |  |
| Yes/No/Not sure | Yes/No/Not sure |
| Please give age when it started (in years) | Open text field |
| 13k. Angina pectoris (chest pains due to lack of oxygen to heart requiring medication such as Glyceryl  Trinitrate sometimes known as GTN)? |  |
| Yes/No/Not sure | Yes/No/Not sure |
| Please give age when it started (in years) | Open text field |
| 13l. Pericarditis or fluid around the heart? |  |
| Yes/No/Not sure | Yes/No/Not sure |
| Please give age when it started (in years) | Open text field |
| 13m. Pericardial constriction (scarring or tightness of the sac around the heart)? |  |
| Yes/No/Not sure | Yes/No/Not sure |
| Please give age when it started (in years) | Open text field |
| 13n. Stiff or leaking heart valves? |  |
| Yes/No/Not sure | Yes/No/Not sure |
| Please give age when it started (in years) | Open text field |
| 13o. Heart catheterisation? |  |
| Yes/No/Not sure | Yes/No/Not sure |
| Please give age when it started (in years) | Open text field |
| 13p. Biopsy of the heart muscle? |  |
| Yes/No/Not sure | Yes/No/Not sure |
| Please give age when it started (in years) | Open text field |
| 13q. Blood clot in head, lung, arm, leg or pelvis? |  |
| Yes/No/Not sure | Yes/No/Not sure |
| Please give age when it started (in years) | Open text field |
| 13r. Does exercise cause severe chest pain, shortness of breath, or irregular heart beat? |  |
| Yes/No/Not sure | Yes/No/Not sure |
| Please give age when it started (in years) | Open text field |
| 13s. Have you seen a cardiologist (heart specialist)? |  |
| Yes/No/Not sure | Yes/No/Not sure |
| Please give age when it started (in years) | Open text field |
| 13t. Has anyone in your immediate family (biological mother, father, brothers, sisters) had a heart attack  before the age of 55? |  |
| Yes/No/Not sure | Yes/No/Not sure |
| Please give age when it started (in years) | Open text field |
| 13u. Any other heart or circulatory problems? |  |
| Yes/No/Not sure | Yes/No/Not sure |
| If yes: please describe | Open text field |
| Please give age when it started (in years) | Open text field |
| **Hearing, vision, speech and taste** |  |
| 14a. Hearing loss requiring a hearing aid? |  |
| Yes/No/Not sure | Yes/No/Not sure |
| Please give age when it started (in years) | Open text field |
| 14b. Deafness in one or both ears not completely corrected by a hearing aid? |  |
| Yes/No/Not sure | Yes/No/Not sure |
| Please give age when it started (in years) | Open text field |
| 14c. Complete deafness in either ear? |  |
| Yes/No/Not sure | Yes/No/Not sure |
| Please give age when it started (in years) | Open text field |
| 14d. Tinnitus or ringing in the ears? |  |
| Yes/No/Not sure | Yes/No/Not sure |
| Please give age when it started (in years) | Open text field |
| 14e. Persistent dizziness or vertigo? |  |
| Yes/No/Not sure | Yes/No/Not sure |
| Please give age when it started (in years) | Open text field |
| 14f. Problems hearing sounds, words, or language in crowds? |  |
| Yes/No/Not sure | Yes/No/Not sure |
| Please give age when it started (in years) | Open text field |
| 14g. Any other hearing problems? |  |
| Yes/No/Not sure | Yes/No/Not sure |
| If yes: please describe | Open text field |
| Please give age when it started (in years) | Open text field |
| 14h. Registered as blind? |  |
| Yes/No/Not sure | Yes/No/Not sure |
| Please give age when it started (in years) | Open text field |
| 14i. Cataracts? |  |
| Yes/No/Not sure | Yes/No/Not sure |
| Please give age when it started (in years) | Open text field |
| 14j. Glaucoma (excess pressure in the eyeball)? |  |
| Yes/No/Not sure | Yes/No/Not sure |
| Please give age when it started (in years) | Open text field |
| 14k. Problems with double vision? |  |
| Yes/No/Not sure | Yes/No/Not sure |
| Please give age when it started (in years) | Open text field |
| 14l. A detached retina or any other condition of the retina? |  |
| Yes/No/Not sure | Yes/No/Not sure |
| If yes: please describe | Open text field |
| Please give age when it started (in years) | Open text field |
| 14m. Any other trouble seeing with one or both eyes even when wearing glasses? |  |
| Yes/No/Not sure | Yes/No/Not sure |
| Please give age when it started (in years) | Open text field |
| 14n. Very dry eyes requiring eye drops or ointment? |  |
| Yes/No/Not sure | Yes/No/Not sure |
| Please give age when it started (in years) | Open text field |
| 14o. Short-sightedness (Myopia) |  |
| Yes/No/Not sure | Yes/No/Not sure |
| Please give age when it started (in years) | Open text field |
| 14p. Long-sightedness (Hypermetropia) |  |
| Yes/No/Not sure | Yes/No/Not sure |
| Please give age when it started (in years) | Open text field |
| 14q. Any other eye problems? |  |
| Yes/No/Not sure | Yes/No/Not sure |
| If yes: please describe | Open text field |
| Please give age when it started (in years) | Open text field |
| 14r. Stammering or stuttering? |  |
| Yes/No/Not sure | Yes/No/Not sure |
| Please give age when it started (in years) | Open text field |
| 14s. Any other speech defects? |  |
| Yes/No/Not sure | Yes/No/Not sure |
| If yes: please describe | Open text field |
| Please give age when it started (in years) | Open text field |
| 14t. Abnormal sense of taste? |  |
| Yes/No/Not sure | Yes/No/Not sure |
| Please give age when it started (in years) | Open text field |
| 14u. Loss of taste or smell which lasted for 3 months or more? |  |
| Yes/No/Not sure | Yes/No/Not sure |
| If yes: please describe | Open text field |
| Please give age when it started (in years) | Open text field |
| **It is important to know whether some forms of medical treatment in childhood affect a man’s potential to father a child.** |  |
| 15a. Has a doctor ever told you that you might have problems fathering a child? |  |
| Yes/No/Not sure | Yes/No/Not sure |
| Please give age when it started (in years) | Open text field |
| 15b. Have you ever had medical tests (such as a blood test, ultrasound or sperm count) to see whether or  not you might have problems fathering a child? |  |
| Yes/No/Not sure | Yes/No/Not sure |
| Please give age when it started (in years) | Open text field |
| 15c. Have you ever been told that you have a low sperm count? |  |
| **16. please indicate if you have ever had any of the following types of surgery and give your approximate age when you first had each type of operation. Please also indicate which types of surgery you never had by ticking the no box.** |  |
| 16a. Amputation of an arm, leg, hand, foot, finger or toe? |  |
| Yes/No/Not sure | Yes/No/Not sure |
| If yes: please describe | Open text field |
| Please give age when it started (in years) | Open text field |
| 16b. Scoliosis surgery (insertion of rods or other methods to straighten the spine)? |  |
| Yes/No/Not sure | Yes/No/Not sure |
| Please give age when it started (in years) | Open text field |
| 16c. Other surgery of your spinal cord or spine? |  |
| Yes/No/Not sure | Yes/No/Not sure |
| If yes: please describe | Open text field |
| Please give age when it started (in years) | Open text field |
| 16d. Leg lengthening or shortening operations? |  |
| Yes/No/Not sure | Yes/No/Not sure |
| Please give age when it started (in years) | Open text field |
| 16e. Joint replacement operations? |  |
| Yes/No/Not sure | Yes/No/Not sure |
| If yes: please describe | Open text field |
| Please give age when it started (in years) | Open text field |
| 16f. Other bone surgery? |  |
| Yes/No/Not sure | Yes/No/Not sure |
| Please give age when it started (in years) | Open text field |
| 16g. Coronary artery bypass surgery? |  |
| Yes/No/Not sure | Yes/No/Not sure |
| Please give age when it started (in years) | Open text field |
| 16h. Pericardiectomy (stripping of the sac around the heart)? |  |
| Yes/No/Not sure | Yes/No/Not sure |
| Please give age when it started (in years) | Open text field |
| 16i. Angioplasty (enlarging a heart vessel using a balloon)? |  |
| Yes/No/Not sure | Yes/No/Not sure |
| Please give age when it started (in years) | Open text field |
| 16j. Other heart surgery? |  |
| Yes/No/Not sure | Yes/No/Not sure |
| If yes: please describe | Open text field |
| Please give age when it started (in years) | Open text field |
| 16k. Surgery for intestinal obstruction (blocked intestines)? |  |
| Yes/No/Not sure | Yes/No/Not sure |
| Please give age when it started (in years) | Open text field |
| 16l. Colostomy or ileostomy (stool going into a bag)? |  |
| Yes/No/Not sure | Yes/No/Not sure |
| Please give age when it started (in years) | Open text field |
| 16m. Reconnection after a colostomy or ileostomy? |  |
| Yes/No/Not sure | Yes/No/Not sure |
| Please give age when it started (in years) | Open text field |
| 16n. Surgery to remove a blood clot in an artery or vein? |  |
| Yes/No/Not sure | Yes/No/Not sure |
| If yes: please describe | Open text field |
| Please give age when it started (in years) | Open text field |
| 16o. Removal of the thyroid gland in your neck? |  |
| Yes/No/Not sure | Yes/No/Not sure |
| Please give age when it started (in years) | Open text field |
| 16p. Removal of your spleen? |  |
| Yes/No/Not sure | Yes/No/Not sure |
| Please give age when it started (in years) | Open text field |
| 16q. Ventriculoperitoneal shunt (tube from the brain to the abdomen, under the skin, which removes excess spinal fluid)? |  |
| Yes/No/Not sure | Yes/No/Not sure |
| Please give age when it started (in years) | Open text field |
| 16r. Breast surgery for removal or biopsy of a suspicious lump? |  |
| Yes/No/Not sure | Yes/No/Not sure |
| Please give age when it started (in years) | Open text field |
| 16s. A bronchoscopy since your therapy stopped? |  |
| Yes/No/Not sure | Yes/No/Not sure |
| Please give age when it started (in years) | Open text field |
| 16t. Other lung surgery? |  |
| Yes/No/Not sure | Yes/No/Not sure |
| If yes: please describe | Open text field |
| Please give age when it started (in years) | Open text field |
| 16u. A liver biopsy since your therapy stopped? |  |
| Yes/No/Not sure | Yes/No/Not sure |
| Please give age when it started (in years) | Open text field |
| 16v. Reconstructive surgery (surgery to repair damage due to an accident or medical therapy or other surgery)? |  |
| Yes/No/Not sure | Yes/No/Not sure |
| If yes: please describe | Open text field |
| Please give age when it started (in years) | Open text field |
| 16w. A heart transplant? |  |
| Yes/No/Not sure | Yes/No/Not sure |
| Please give age when it started (in years) | Open text field |
| 16x. A lung transplant? |  |
| Yes/No/Not sure | Yes/No/Not sure |
| Please give age when it started (in years) | Open text field |
| 16y. A kidney transplant? |  |
| Yes/No/Not sure | Yes/No/Not sure |
| Please give age when it started (in years) | Open text field |
| 16z. A bone marrow transplant? |  |
| Yes/No/Not sure | Yes/No/Not sure |
| Please give age when it started (in years) | Open text field |
| 17a. Other organ transplant? |  |
| Yes/No/Not sure | Yes/No/Not sure |
| If yes: please describe | Open text field |
| Please give age when it started (in years) | Open text field |
| 17b. Cataract surgery? |  |
| Yes/No/Not sure | Yes/No/Not sure |
| Please give age when it started (in years) | Open text field |
| 17c. Sinus surgery? |  |
| Yes/No/Not sure | Yes/No/Not sure |
| Please give age when it started (in years) | Open text field |
| 17d. Surgery on your jaw? |  |
| Yes/No/Not sure | Yes/No/Not sure |
| Please give age when it started (in years) | Open text field |
| 17e. Any other surgery? |  |
| Yes/No/Not sure | Yes/No/Not sure |
| If yes: please describe | Open text field |
| Please give age when it started (in years) | Open text field |
| **18a. Did your childhood cancer, leukaemia, tumour or similar illness ever come back (recur or relapse)**  **after it was first treated?** |  |
| Yes/No | Yes/No |
| If yes, please give details of the first time it came back after the first treatment |  |
| - Date doctor confirmed illness has come back | DD/MM/YYYY |
| - Doctor’s name | Open text field |
| - Hospital name | Open text field |
| - Hospital address | Open text field |
| **18b. Apart from your original childhood cancer, leukaemia, tumour or similar illness, have you ever**  **been diagnosed with any OTHER cancer, leukaemia or tumour?** |  |
| Yes/No | Yes/No |
| If yes, please give details of the first time it came back after the first treatment |  |
| - Date doctor confirmed illness has come back | Open text field |
| - Illness diagnosed | DD/MM/YYYY |
| - Doctor’s name | Open text field |
| - Hospital name | Open text field |
| - Hospital address | Open text field |

^A^ Question no. 11.b was numbered twice in the original British Childhood Cancer Survivor Study questionnaire.

**Supplementary Table S4**. Summary of all outcomes embedded in the three selected questionnaires

This table summarizes all outcomes that were embedded in the three selected questionnaires (the Dutch Childhood Oncology – Long-Term Effects After Childhood Cancer (DCOG-LATER) study questionnaire, the U.S.A.-based Childhood Cancer Survivor Study (CCSS) questionnaire and the British Childhood Cancer Survivor Study (BCCSS) questionnaire), for 15 main outcome categories.

The X’s represent health conditions that are embedded in the respective questionnaire.

|  | **DCOG LATER** | **CCSS** | **BCSS** | **Concordant discordant** | **Present in the draft outcome list (yes/no)^A^** |
| --- | --- | --- | --- | --- | --- |
| **Eye** |  |  |  |  |  |
| Cataract | X | X | X | Concordant | Yes |
| Blind |  | X (registered as blind) | X (registered as blind) | Discordant | Yes |
| Glaucoma |  | X | X | Discordant | No |
| Double vision |  | X | X | Discordant | No |
| Detached retina |  | X | X | Discordant | No |
| Other retina condition |  | X | X | Discordant | No |
| (very) Dry eyes |  | X | X | Discordant | No |
| Other trouble seeing with one or both eyes |  | X | X | Discordant | No |
| Myopia |  | X | X | Discordant | No |
| Hypermetropia |  | X | X | Discordant | No |
| Other eye problems |  | X | X | Discordant | No |
| **Ear** |  |  |  |  |  |
| Hearing loss |  | X (requiring hearing aid) | X (requiring hearing aid) | Discordant | Yes |
| Hearing aid | X |  |  | Discordant | No |
| Deafness |  | X | X | Discordant | Yes |
| Tinnitus | X | X | X | Concordant | No |
| Dizziness |  | X | X | Discordant | No |
| Problems hearing sounds/words/language in crowds |  | X | X | Discordant | No |
| **Speech** |  |  |  |  |  |
| Stammering/stuttering |  | X | X | Discordant | No |
| Other speech defects |  | X | X | Discordant | No |
| Abnormal sense of taste |  | X | X | Discordant | No |
| Loss of taste for 3 months or more |  | X | X | Discordant | No |
| **Cardiac** |  |  |  |  |  |
| Myocardial infarction | X | X | X | Concordant | Yes (Cardiac ischemia) |
| Coronary heart disease |  | X | X | Discordant | No |
| Angina pectoris | X | X | X | Concordant | Yes (Cardiac ischemia) |
| Valvular condition | X | X (stiff or leaking valves) | X (stiff or leaking valves) | Concordant | No |
| Pericarditis | X | X | X | Concordant | No |
| Pericardial constriction |  | X | X |  | No |
| Cardiomyopathy | X | X (congestive heart failure or cardiomyopathy) | X | Concordant | No |
| Heart failure | X | X (congestive heart failure or cardiomyopathy) |  | Concordant | Yes |
| Arrythmia | X | X | X | Concordant | Yes |
| Congenital heart disease | X |  |  | Discordant | No |
| Rheumatic heart disease |  | X | X | Discordant | No |
| Arteriosclerosis |  | X | X | Discordant | No |
| Murmur |  | X | X | Discordant | No |
| Heart catheterisation |  | X | X | Discordant | No |
| Biopsy of heart muscle |  | X | X | Discordant | No |
| Heart failure during pregnancy or after delivery (females) |  | X |  | Discordant | No |
| Other heart disease | X | X | X | Concordant | No |
| Does exercise cause chest pain/shortness of breath/irregular heartbeat |  | X | X | Discordant | No |
| Have you seen a cardiologist |  | X | X | Discordant | No |
| Has anyone in your family had a heart attack before age of 55 |  | X | X | Discordant | No |
| **Vascular** |  |  |  |  |  |
| Vascular conditions in general | X |  |  | Discordant | No |
| Blood clot in lung/arm/leg/pelvis |  | X | X | Discordant | Yes (thrombosis) |
| Hypertension | X | X | X | Concordant | Yes |
| Conditions leading to enhanced coagulation | X |  |  | Discordant | No |
| Hypercholesterolemia | X |  |  | Discordant | No |
| **Pulmonary** |  |  |  |  |  |
| Pulmonary conditions in general | X |  |  | Discordant | No |
| Cough | X | X (chronic cough or shortness of breath for greater than one month) | X (chronic cough or shortness of breath for greater than one month) | Concordant | No |
| Respiratory tract infections over 3 times a year | X |  |  | Discordant | No |
| Recurrent sinus infections |  | X | X | Discordant | No |
| Tonsillitis or enlargement of tonsils or adenoids |  | X | X | Discordant | No |
| Bronchitis |  | X | X | Discordant | No |
| Pneumonia 3 or more times past 2 years |  | X | X | Discordant | No |
| Hay Fever |  | X | X | Discordant | No |
| Pleurisy |  | X | X | Discordant | No |
| Asthma |  | X | X | Discordant | Yes (obstructive pulmonary disease) |
| Abnormal chest wall |  | X | X | Discordant | No |
| Need for extra oxygen |  | X | X | Discordant | No |
| Emphysema |  | X | X | Discordant | No |
| Lung fibrosis |  | X | X | Discordant | No |
| Other breathing problems |  | X | X | Discordant | No |
| **Gastro-intestinal** |  |  |  |  |  |
| Gastro-intestinal conditions in general | X |  |  | Discordant | No |
| Ulcer |  | X | X | Discordant | No |
| Disease of esophagus |  | X | X | Discordant | No |
| Frequent indigestion |  | X | X | Discordant | No |
| Frequent heartburn (medication) |  | X | X | Discordant | Yes (gastro-interstinal reflux) |
| Other stomach trouble |  | X | X | Discordant | No |
| Intestinal polyps |  | X | X | Discordant | No |
| Diverticular disease |  | X | X | Discordant | No |
| Colitis |  | X | X | Discordant | No |
| Drequent constipation |  | X | X | Discordant | No |
| Chronic diarrhea |  | X | X | Discordant | No |
| Rectal or anal fistula |  | X | X | Discordant | No |
| Rectal stricture |  | X | X | Discordant | No |
| Other digestive problems |  |  | X | Discordant | Yes |
| **Liver** |  |  |  |  |  |
| Conditions of the liver in general | X |  |  | Discordant | No |
| Gallstones |  | X | X | Discordant | No |
| Other gallbladder trouble |  | X | X | Discordant | No |
| Cirrhosis of the liver |  | X | X | Discordant | Yes |
| Hepatitis |  | X | X | Discordant | Yes |
| Jaundice |  | X | X | Discordant | No |
| Other liver trouble |  | X | X | Discordant | No |
| **Renal and urinary tract** |  |  |  |  |  |
| Renal and urinary tract conditions in general | X |  |  | Discordant | No |
| Pyelonefritis | X | X (repeated kidney infections) | X (repeated kidney infections) | Concordant | No |
| Repeated bladder infections |  | X | X | Discordant | No |
| Kidney stones |  | X | X | Discordant | No |
| Dialysis |  | X | X | Discordant | No |
| Other kind of kidney or urinary tract disorder |  | X | X | Discordant | No |
| **Endocrine** |  |  |  |  |  |
| Adrenal problems | X |  |  | Discordant | Yes |
| Diabetes mellitus | X | X (diet/pills/insulin injections) | X (diet/pills/insulin injections) | Concordant | Yes |
| Lack of growth hormone |  | X | X | Discordant | Yes |
| Injections of growth hormone |  | X | X | Discordant | No |
| Short stature | X |  |  | Discordant | No |
| Hypothyroidism | X | X | X | Concordant | Yes |
| Hyperthyroidism | X | X | X | Concordant | Yes |
| Thyroid nodule | X | X | X | Concordant | No |
| Other thyroid Enlargement |  | X | X | Discordant | No |
| Other thyroid problem | X |  |  | Discordant | No |
| Osteoporosis |  | X | X | Discordant | No |
| Medication to go into puberty |  | X | X | Discordant | Yes |
| Problems with fertility |  | X | X | Discordant | No |
| Has a doctor told you might have trouble having children |  | X | X | Discordant | No |
| Have you ever had medical tests to see whether you might have trouble having children |  | X | X | Discordant | No |
| Have you ever been told you have a low sperm count (males) |  | X | X | Discordant | No |
| Have you ever had a menstrual period (female) |  | X | A | Discordant | No |
| Are you currently having menstrual periods (female) |  | X | A | Discordant | No |
| Have you ever taken female hormones including birth control pills to have your period (female) |  | X | A | Discordant | No |
| Other problem with hormonal regulation | X | X | X | Concordant | No |
| **Musculoskeletal** |  |  |  |  |  |
| Musculoskeletal problems in general | X |  |  | Discordant | Yes |
| **Neurological** |  |  |  |  |  |
| Stroke | X (Cerebrovascular accident, Cerebral infarction) | X (classified as heart and circulatory system) | X (classified as heart and circulatory system) | Concordant | Yes |
| Transient Ischemic Attack | X |  |  | Discordant | Yes |
| Seizures | X | X | X | Concordant | No |
| Epilepsy |  | X | X | Discordant | Yes |
| Cerebral palsy |  | X | X | Discordant | No |
| Paralysis of any kind |  | X | X | Discordant | No |
| Mental retardation |  | X | X | Discordant | No |
| Migraine |  | X | X | Discordant | No |
| Headache |  | X | X | Discordant | Yes |
| Problems with balance |  | X | X | Discordant | No |
| Tremors |  | X | X | Discordant | No |
| Weakness |  | X | X | Discordant | No |
| Decreased sense/touch |  | X | X | Discordant | No |
| Prolonged pain |  | X | X | Discordant | No |
| Problems chewing/swallowing |  | X | X | Discordant | No |
| Other brain or nervous system problem |  | X | X | Discordant | No |
| Nervousness |  | X |  | Discordant | No |
| Faintness or dizziness |  | X |  | Discordant | No |
| Pains in heart or chest |  | X |  | Discordant | No |
| Thoughts on ending your life |  | X |  | Discordant | No |
| Suddenly scared for no reason |  | X |  | Discordant | No |
| Feeling lonely |  | X |  | Discordant | No |
| Feeling blue |  | X |  | Discordant | No |
| Feeling to interest in things |  | X |  | Discordant | No |
| Feeling fearful |  | X |  | Discordant | No |
| Feelings being easily hurt |  | X |  | Discordant | No |
| Nausea or upset stomach |  | X |  | Discordant | No |
| Trouble getting your breath |  | X |  | Discordant | No |
| Hot or cold spells |  | X |  | Discordant | No |
| Numbness or tingling in parts of your body |  | X |  | Discordant | No |
| Feeling hopeless about the future |  | X |  | Discordant | No |
| Feeling weak in parts of your body |  | X |  | Discordant | No |
| Feeling tense or keyed up |  | X |  | Discordant | No |
| Spells of terror or panic |  | X |  | Discordant | No |
| Feeling so restless you couldn’t sit at all |  | X |  | Discordant | No |
| Feelings of worthlessness |  | X |  | Discordant | No |
| Pain |  | X |  | Discordant | No |
| Anxieties |  | X |  | Discordant | No |
| **Surgical procedures** |  |  |  |  |  |
| Coronary artery bypass surgery |  | X | X | Discordant | No |
| Pericardectomy |  | X | X | Discordant | No |
| Angioplasty |  | X | X | Discordant | No |
| Replacement of heart valve | X |  |  | Discordant | No |
| Surgery to remove a blood clot in an artery or vein |  | X | X | Discordant | No |
| Pacemaker/ICD | X |  |  | Discordant | No |
| Other heart surgery | X | X | X | Concordant | No |
| Removal of organ | X |  |  | Discordant | Yes |
| Removal of arm/leg | X | X (also only hand/foot) | X (also only hand/foot/finger/toe) | Concordant | Yes |
| Scoliosis surgery |  | X | X | Discordant | No |
| Other surgery to the spinal cord or spine |  |  | X | Discordant | No |
| Leg lengthening or shortening procedures |  | X | X | Discordant | No |
| Joint replacement |  | X | X | Discordant | No |
| Other bone surgery |  | X | X | Discordant | No |
| Surgery for intestinal obstruction |  | X | X | Discordant | No |
| Colostomy |  | X | X (or ileostomy) | Discordant | No |
| Takedown of the colostomy or ileostomy |  | X | X | Discordant | No |
| Removal of the thyroid gland in the neck |  | X | X | Discordant | No |
| Removal of the spleen |  | X | X | Discordant | Yes |
| Ventriculoperitoneal shunt |  | X | X | Discordant | No |
| Breast surgery |  | X | X | Discordant | No |
| Bronchoscopy |  | X | X | Discordant | No |
| Other lung surgery |  | X | X | Discordant | No |
| Liver biopsy |  | X | X | Discordant | No |
| Reconstructive surgery |  | X | X | Discordant | No |
| Heart transplant |  | X | X | Discordant | Yes |
| Lung transplant |  | X | X | Discordant | Yes |
| Kidney transplant |  | X | X | Discordant | Yes |
| Bone marrow transplant |  | X | X | Discordant | No |
| Organ transplantation (other) | X | X | X | Concordant | No |
| Cataract surgery |  | X | X | Discordant | No |
| Sinus surgery |  | X | X | Discordant | No |
| Surgery on the jaw |  | X | X | Discordant | No |
| Other surgery |  | X | X | Discordant | No |
| **Other** |  |  |  |  |  |
| Other conditions in general | X |  |  | Discordant | No |
| **Cancer, leukemia or tumors** |  |  |  |  |  |
| Cancer, leukemia or tumors | X | X | X | Concordant | Yes |

^A^ only male list available online.

**Supplementary Table S5.** Core set of long-term self-reported physical outcomes that are clinically relevant for CCS, and correspondence to Common Terminology Criteria of Adverse Events (CTCAE) severity grading.

|  | **Long-term self-reported physical outcome** | **Minimum corresponding CTCAE grade** | **Minimum corresponding SJLIFE grade** |
| --- | --- | --- | --- |
| **Eye** | Cataract | 3 (max 4) ^A^ | 3 (max 4) |
| **conditions** | Blindness | ^B^  Listed as outcome (grade 4) of multiple “Eye disorders” | B |
|  | Eye removal | B | B |
| **Ear** | Hearing loss | 3 | C |
| **conditions** | Deafness | B | B |
| **Cardiac** | Heart failure | 3 | 3 |
| **conditions** | Ischemia | 3  Listed as “Acute coronary syndrome” | 3  Listed as “Coronary artery disease) |
|  | Periarditis | 3 | 3 |
|  | Valvular disease | 3 | 3 |
|  | Arrythmia | 2 | 2 |
|  | Heart transplantation | B | B |
| **Vascular** | Hypertension | 2 | 2 |
| **conditions** | Thrombosis | 2 | 2 |
|  | Aneurysm | B | 3-4 |
| **Respiratory conditions** | Obstructive pulmonary disease | 2 Listed as “Bronchospasm” | 2 (for COPD)  3 (for asthma) |
|  | Decreased pulmonary function | ^C^ (max 3)  Listed under “Investigations” as “Carbon monoxide diffusing capacity decreased”, grading based on carbon monoxide diffusing capacity, all grades are asymptomatic | C |
|  | Pulmonary resection | B | B |
|  | Pulmonary transplantation | B | B |
|  | Other pulmonary conditions | 2 | B |
| **Gastro-intestinal** | Gastroesophageal reflux disease | 2 (max 3)^A^ | 2 (max 3) |
| **conditions** | Inflammatory bowel disease | 4  Listed as “Enterocolitis” | 4  Listed as “Enterocolitis” |
|  | Other gastrointestinal conditions | 2 | B |
| **Hepatobiliary** | Hepatitis | 3  Listed under “Infections” | 2  Listed under “Infections” |
| **conditions** | Hemochromatosis | 3  Listed under “Metabolism and nutrition disorders” as “Iron overload” | 2  Listed under “Hematologic” |
|  | Liver cirrhosis | ^B^  Listed as outcome (grade 3) of “Viral hepatitis” | C |
|  | Liver transplantation | B | B |
|  | Cholecystectomy | B | B |
| **Renal and** | Tubular dysfunction | B | B |
| **urinary tract conditions** | Proteinuria | ^C^ (max 3)^A^  Grading based on urinary protein level, no separate grade for treatment required | B |
|  | Chronic kidney disease | ^C^ Grading based on eGFR or CrCl, no separate grade for treatment required | 2 |
|  | Urinary tract obstruction | 2 | 2 |
|  | Nephrectomy | B | B |
|  | Renal transplantation | ^B^  Listed as outcome of chronic kidney disease (grade 4) | ^B^  Listed as outcome of chronic kidney disease (grade 4) |
|  | Other conditions of kidney and urinary tract | 2 | B |
| **Endocrine** | Adrenal insufficiency^X^ | 2 | 1 |
| **conditions** | Hypercortisolism | 2 (max 3)^A^ | B |
|  | Hypothyroidism^X^ | 2 | 2 |
|  | Hyperthyroidism | 2 | 2 |
|  | Estrogen deficiency^X^ | 2 Not listed as a separate condition; “other endocrine conditions” | B |
|  | Testosterone deficiency^X^ | 2 Not listed as a separate condition; “other endocrine conditions” | B |
|  | Growth hormone deficiency^X^ | 2 (max 2)^A^ Listed under “Investigations” as “Growth hormone abnormal” | 1 (max 2) |
|  | Hypoparathyroidism | 2 | 2 |
|  | Hyperparathyroidism | 2 (max 2)^A^ | 2 (max 2) |
|  | Prolactinoma | B | 2  Listed as “hyperprolactinemia” |
|  | Polycystic ovarian syndrome | B | 2 |
|  | Pubertas praecox | ^C^  Grading based on biochemical markers and age, not a separate grade for treatment required (max 2) | ^C^  Grading based on biochemical markers and age, not a separate grade for treatment required (max 2) |
|  | Pubertas tarda | 3 (max 3) | B |
|  | Pituitary deficiency | B | B |
|  | Pituitary surgery | B | B |
|  | Obesity | 3 (max 4) Listed under “Metabolism and nutrition disorders” | 3 (max 4) |
|  | Underweight | B | 2 (max 2) |
|  | Diabetes mellitus | ^C^  “Hyperglycaemia” listed under “Metabolism and nutrition disorders”. Grading based on fasting glucose levels and hospitalization indication, no separate grade for treatment required | 2 |
|  | Diabetes insipidus^X^ | 2 Not listed as a separate condition; “other endocrine conditions” | 2 |
|  | Thyroidectomy | B | B |
|  | Adrenal gland removal | B | B |
|  | Ovariectomy | B | B |
|  | Orchidectomy | B | B |
| **Nervous system conditions** | Cerebrovascular accident – hemorrhagic | 2  Listed under “Nervous system disorders” as “Intracranial hemorrhage”. Also listed under “Nervous system disorders” as “Stroke”, grading based on neurologic deficit with no separate grade for treatment required | 2 |
|  | Cerebrovascular accident – ischemic | ^C^  Listed under “Nervous system disorders” as “Stroke” or as “Cerebrovascular ischemia” (max 2), grading based on neurologic deficit with no separate grade for treatment required | 2 |
|  | Transient ischemic attack | ^C^ (max 2)  Grading based on neurologic deficit and imaging, no separate grade for treatment required | B |
|  | Epilepsy | 2-3^C^  Listed as “Seizure”, grade 2 is brief generalized seizure and grade 3 is multiple seizures despite medical intervention | 2 |
|  | Headache | ^C^ (max 3)  Grading based on intensity of pain and ADL, no separate grade for treatment required | 3 |
|  | Hydrocephalus | 3 | 3 |
|  | Other neurological conditions | 2 | B |
| **Mulculoskeletal** | Amputation | B | 2 (max 3) |
| **conditions** | Deformities | 2 (max 3)^A^ | 2 (max 3) |
|  | Osteoporosis | 2 (max 3)^A^ | C |
|  | Other musculoskeletal conditions | 2 | B |
| **Neoplasms** | Malignant neoplasms | 2 | B |
| **Other conditions** | Dermatological conditions | 2-3 | B |
|  | Hysterectomy | B | B |
|  | Prostatectomy | B | B |
|  | Mastectomy | B | B |
|  | Splenectomy | B | B |

^A^: (max) is stated when the maximum possible CTCAE grade is below grade 5. In this case, the number at (max) represents the maximum possible CTCAE grade for that condition is shown.

^B^: We could not perform CTCAE grading, because the condition was not present in the CTCAE as a separate entity.

^C^: It was not possible to define the corresponding CTCAE grade for our established clinical relevance criteria, because additional clinical information was needed for CTCAE-based grading.

^X^: When this hormonal deficiency is the result of pituitary dysfunction, it is categorized separately as “Pituitary deficiency”.
